# Evaluation of Different Chemotypes of *Lavandula angustifolia* Essential Oils as Phytobiotics for the Control of Clinical *Escherichia coli* Strains Isolated from Commercial Poultry Production

**DOI:** 10.3390/molecules31193495

**Published:** 2026-10-01

**Authors:** Michalina Adaszyńska-Skwirzyńska, Małgorzata Dzięcioł, Sławomir Zych

**Affiliations:** 1 Department of Monogastric Animal Sciences, Faculty of Biotechnology and Animal Sciences, West Pomeranian University of Technology in Szczecin, Janickiego St. 29, 71-270 Szczecin, Poland; madaszynska@zut.edu.pl; 2 Department of Organic Chemical Technology and Polymer Materials, Faculty of Chemical Technology and Engineering, West Pomeranian University of Technology in Szczecin, Piastów Ave. 42, 71-065 Szczecin, Poland; 3 Department of Microbiology and Biotechnology, Faculty of Biotechnology and Animal Sciences, West Pomeranian University of Technology in Szczecin, Piastów Ave. 45, 70-310 Szczecin, Poland; slawomir.zych@zut.edu.pl

**Keywords:** *Lavandula angustifolia*, essential oils, phytobiotics, *Escherichia coli*, antibacterial activity, antibiotic resistance, colibacillosis in poultry

## Abstract

The study focused on evaluating the antibacterial activity of ten essential oils (EOs) representing different origins and chemotypes of common lavender (*Lavandula angustifolia*), as well as their two major constituents, linalool and linalyl acetate. The aim of the study was to evaluate their potential as natural phytobiotics against clinical *Escherichia coli* strains isolated from poultry, including multidrug-resistant avian pathogenic *E. coli* as well as the reference strain ATCC 25922. GC-MS analysis demonstrated variation in the chemical composition of the EOs, with linalool (16.00–35.95%) and linalyl acetate (18.15–43.61%) identified as the major constituents. Determination of the minimum inhibitory concentration (MIC) by the broth microdilution method showed that pure linalool exhibited the strongest antibacterial activity (mean MIC = 0.31% *v*/*v*), followed by the EO from the lavender variety ‘Blue River’ (0.85% *v*/*v*), and the varieties ‘Maillette’ and ‘Ellagance Purple’ (0.98% and 1.00% *v*/*v*, respectively). Linalyl acetate had 5-fold lower antibacterial activity than linalool (1.63% *v*/*v*), whereas the weakest activity was observed for the variety ‘Matherone’ EO (4.50% *v*/*v*). Statistical analysis confirmed a significant correlation between higher linalool content and stronger antibacterial activity of the tested EOs against various strains of *E. coli*. Time–kill analysis showed that linalool and EOs with high linalool levels completely eliminated the multidrug-resistant *E. coli* strain within 4–8 h. EOs from *L. angustifolia* show potential as an alternative to conventional antibiotics in poultry production but their efficacy is highly dependent on the chemotype.

## 1. Introduction

Avian pathogenic *Escherichia coli* (APEC) poses a major threat to the poultry industry, causing colibacillosis, a complex disease characterized by multisystemic lesions and high mortality, particularly in young birds [1]. It is estimated that approximately 10–15% of *E. coli* strains present in the avian intestinal tract belong to APEC serotypes [2]. These pathogens are a major source of economic losses in commercial poultry production [3,4]. Colibacillosis in poultry is primarily associated with *E. coli* strains belonging to the O78:K80, O1:K1, O2:K1, and O18:K1 serotypes. APEC strains are phenotypically and genotypically highly similar to commensal *E. coli* strains [5]. The disease is characterized by multisystemic lesions associated with conditions including hemorrhagic septicemia, swollen head syndrome, airsacculitis (chronic respiratory disease), cellulitis, peritonitis, osteomyelitis, synovitis, yolk sac infection, enteritis, pharyngitis, colisepticemia, and coligranuloma [2,6,7]. In newly hatched chicks, *E. coli* infection may originate from colonization of the pharynx or infection of the navel and/or yolk sac, both of which are likely to progress to septicemia [8]. These pathological changes account for approximately 50% of overall mortality during the first week of the chick’s life [9]. Moreover, APEC are recognized as a potential reservoir of antimicrobial resistance genes that may be transferred to human pathogens, representing a global public health challenge in the One Health initiative [10]. This concept recognizes that human, animal, and environmental health are closely interconnected, and that antimicrobial resistance encompasses interrelated issues involving humans, livestock, and antibiotic residues in food and the environment [10].

In avian pathogenic *E. coli*, antimicrobial resistance is an increasing concern, as 97% of isolates from poultry farms worldwide are resistant to at least one antimicrobial agent, while 40% show resistance to at least three antimicrobial agents [11,12]. This represents a major public health concern because these strains can transfer antimicrobial resistance genes to other microorganisms within the human intestinal microbiota. When these strains come into contact with other microorganisms capable of acquiring resistance genes from APEC through horizontal gene transfer, the widespread occurrence of antimicrobial resistance makes the transmission of resistance genes, including to pathogenic strains, highly likely, thereby significantly reducing the effectiveness of conventional antimicrobial therapy [13].

The increasing antimicrobial resistance, caused by the overuse of antibiotics in animal production, has made the effective eradication of pathogens increasingly difficult and shows the need for alternative strategies to control infections [14]. Plant secondary metabolites, such as essential oils (EOs), are among the promising alternatives as they possess broad-spectrum antibacterial activity [15]. A good example of an aromatic plant with antibacterial properties is narrow-leaved (common) lavender (*L. angustifolia*), known for EOs containing a wide range of biologically active compounds [16,17]. The biological activity of EOs depends on their chemical composition and the relative content of their main constituents [18,19]. The chemical composition of lavender essential oil, however, is complex and depends on several factors, including variety, geographical origin, cultivation conditions, and the isolation method [20,21,22,23]. Reports on the antibacterial activity of lavender EOs against various bacteria can be found in the literature, including promising results regarding *E. coli* [16,18,22], but the available data pertain only selected single bacterial strains. Therefore, the objective of this study was to evaluate the antibacterial activity of EOs from *L. angustifolia* obtained from different cultivation sources worldwide, as well as their major chemical components (linalool and linalyl acetate), against avian colibacillosis, using a large collection of 19 clinical *E. coli* isolates with different antimicrobial susceptibility profiles and the reference *E. coli* strain ATCC 25922 (a human, antibiotic-susceptible strain).

## 2. Results

### 2.1. Chemical Composition of Lavender Essential Oils

The study included 10 essential oils from *L. angustifolia* varieties originating from various geographical regions, resulting in differences in their chemical composition. Table 1 summarizes the available information on the EOs tested, including *L. angustifolia* variety, geographical origin, producer, and the code assigned to each EO in the present study.

GC-MS analysis revealed significant differences in the chemical composition of *L. angustifolia* EOs. A total of 34 compounds present at concentrations above 0.1% were identified in the EOs (Table 2). A common characteristic of all EOs was that linalool (16.00–35.95%) and linalyl acetate (18.15–43.61%) were the dominant constituents in all samples. The highest linalool content (>30%) was detected in the EOs from the variety *L. angustifolia* ‘Ellagance Purple’ (ELP) and ‘Maillette’ (MAIL), whereas the lowest content (16%) was observed in the EO from the variety ‘Matherone’ (MATH). In contrast, the highest linalyl acetate content (approximately 40% or higher) was found in the EOs from the *L. angustifolia* varieties ‘Kashmir’ (KASH) and ‘Matherone’ (MATH), whereas the lowest content (18.15%) was observed in the EO from variety ‘Ellagance Purple’ (ELP). Some EOs also contained relatively high levels of lavandulyl acetate, β-caryophyllene, terpinen-4-ol, α-terpineol, *trans*-β-farnesene, and *cis*- or *trans*-β-ocimene; however, their concentrations did not exceed 10% for lavandulyl acetate or 8% for the remaining compounds. It should also be noted that some of the EOs contained unique compounds characteristic of specific *L. angustifolia* varieties. Examples include cryptone (1.84%), detected exclusively in BLR; α-santalene (2.64%), which was also detected in AVI, but at a much lower level (0.9%); and pinocarveol (0.76%), found exclusively in ELP.

**Figure 1:**
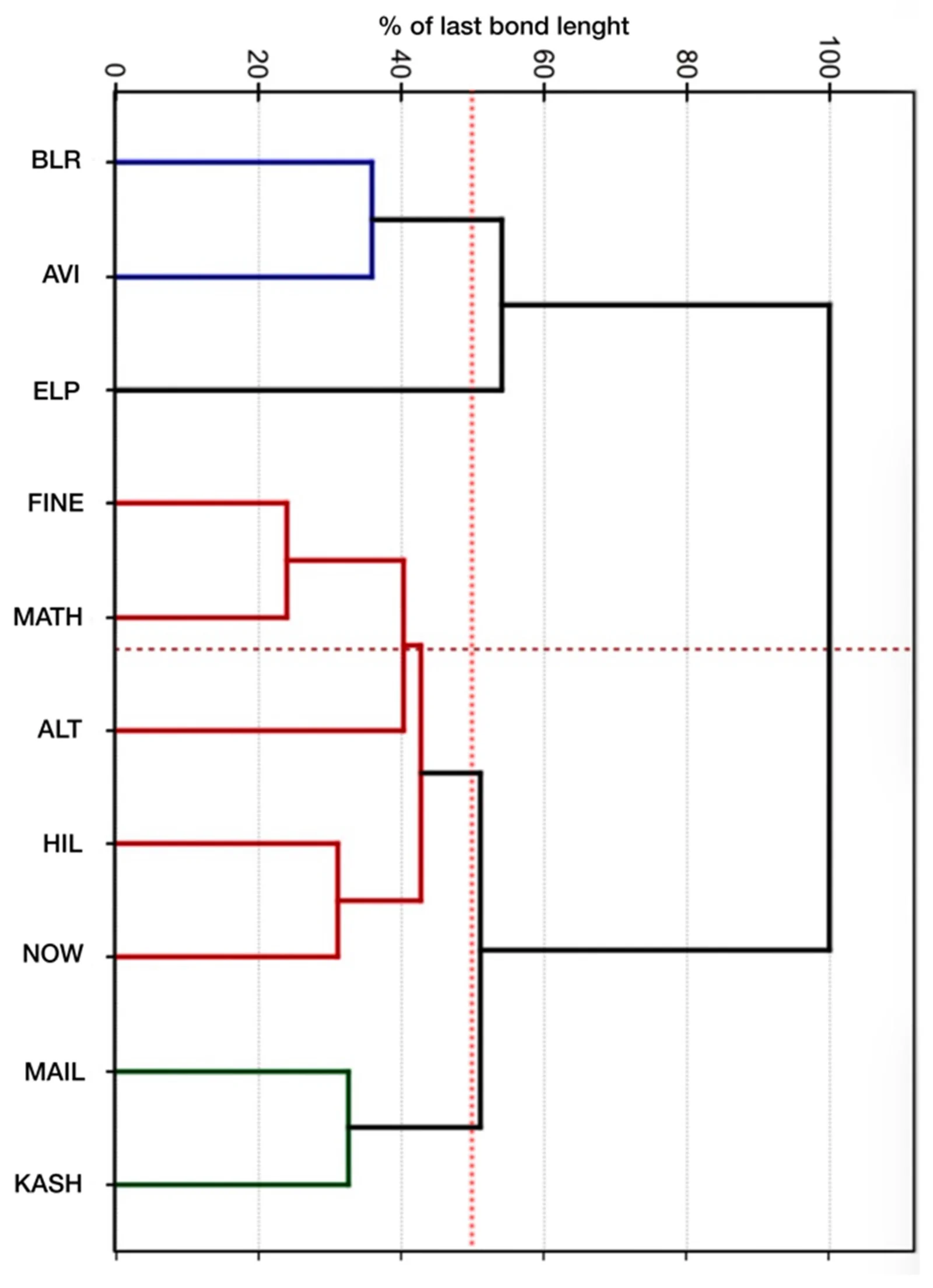
Hierarchical cluster analysis of *Lavandula angustifolia* essential oils.

To compare the chemical composition of the EOs, GC-MS data were subjected to hierarchical cluster analysis using Ward’s method with Euclidean distance. Figure 1 shows the results of the hierarchical cluster analysis, illustrating the similarities and differences between the 10 studied *L. angustifolia* EOs based on their chemical composition. Analysis of the dendrogram identified four main EO clusters with a high degree of similarity between their members. The first cluster comprised two EOs, BLR from Poland and AVI from Bulgaria. The second cluster, closely related to the first, consisted solely of ELP, also obtained from lavender cultivated in Poland. The third and largest cluster contained five EOs originating from France, Bulgaria, and Greece: FINE, MATH, ALT, HIL, and NOW. Within this cluster, two pairs of closely related EOs could be further distinguished: FINE and MATH, and HIL and NOW. The fourth cluster contained MAIL from France and KASH from India, forming a distinct subgroup that was relatively closely related to the third cluster. The dendrogram indicated a high degree of similarity between the following EO pairs: BLR–AVI, FINE–MATH, HIL–NOW, and MAIL–KASH. The results indicate significant variation in EO chemotypes, which appears to be primarily associated with the lavender variety rather than its geographical origin.

### 2.2. Antimicrobial Activity Against Escherichia coli

The MIC values for 10 lavender EOs and their principal components, linalool and linalyl acetate, assessed against 20 *E. coli* strains, including 10 multidrug-resistant (MDR) and 9 susceptible (S) clinical isolates from commercial poultry farms, as well as the antibiotic-susceptible human reference *E. coli* strain ATCC 25922, are presented in Table 3.

**Figure 2:**
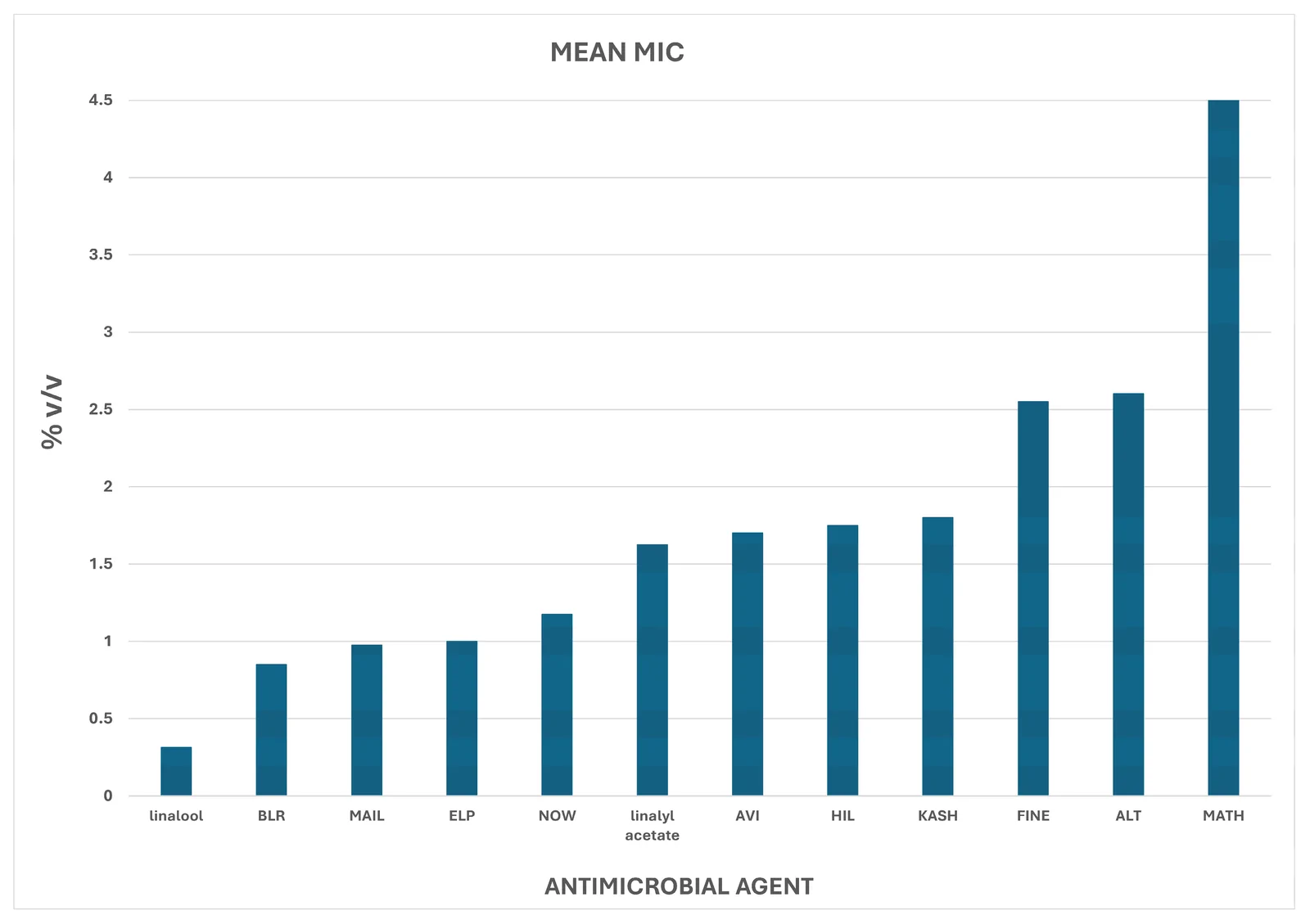
Gradient of the antimicrobial activity of ten lavender essential oils and the two compounds determining their chemotype (linalool and linalyl acetate), based on the mean MIC values.

Pure linalool showed the highest antibacterial activity against all *E. coli* strains (mean MIC, 0.31% *v*/*v*), followed by the BLR EO (mean MIC 0.85% *v*/*v*). High antibacterial activity was also observed for the MAIL and ELP EOs, with mean MICs of 0.98% and 1.00% *v*/*v*, respectively. The NOW EO showed only slightly lower activity, with a mean MIC of 1.18% *v*/*v*. Pure linalyl acetate was 5-fold less active than pure linalool, with a mean MIC of 1.27% *v*/*v*. The next group comprised EOs with comparable antibacterial activity, with mean MIC values ranging from 1.70 to 1.80% *v*/*v* (AVI, HIL, KASH). The remaining EOs had mean MIC values above 2% *v*/*v*: FINE (2.55% *v*/*v*), ALT (2.60% *v*/*v*), and MATH, which showed the lowest antibacterial activity (4.50% *v*/*v*). It is worth noting that MATH, being the least active among the essential oils tested, contained a high proportion of linalyl acetate (39.5%) and the lowest linalool content (16%) of all the EOs. A graphical comparison of the mean MIC values for all studied EOs and their two main components is presented in Figure 2. This allows for an easy comparison of the antimicrobial agents tested, as a lower bar on a graph indicates generally stronger activity against the various strains of *E. coli*.

**Figure 3:**
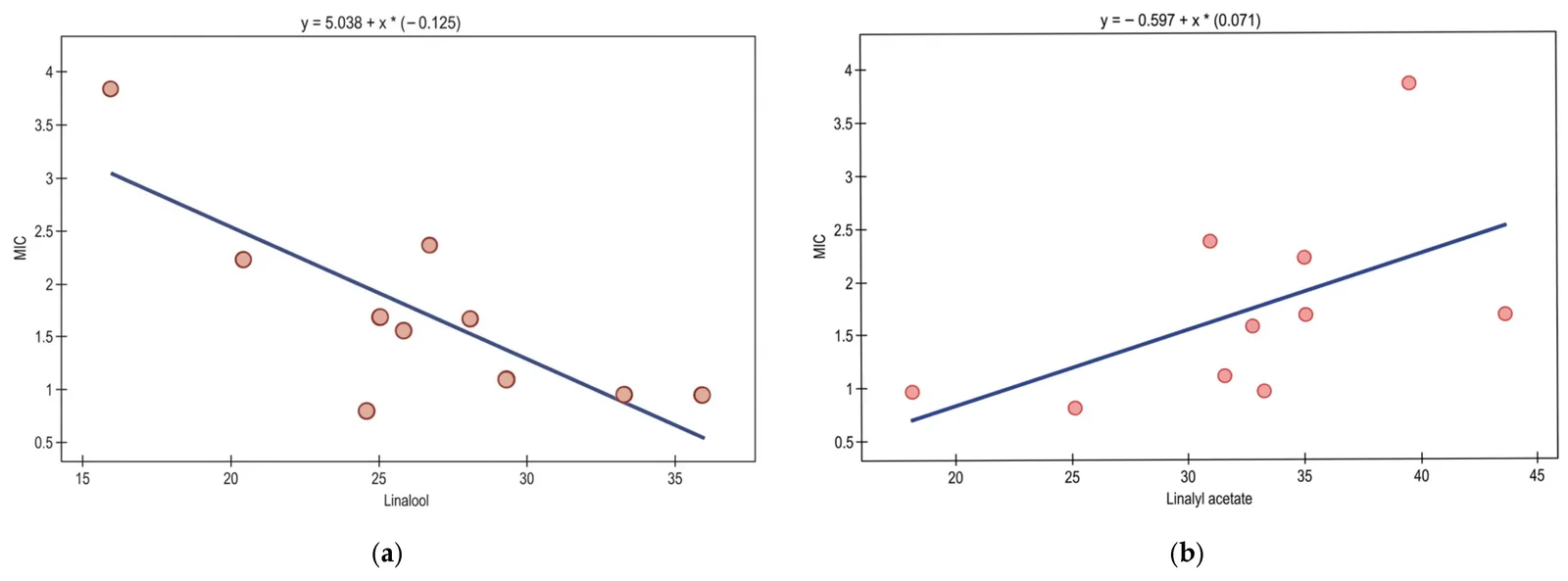
Pearson’s linear correlation between the relative content [%] of linalool (**a**) or linalyl acetate (**b**) and the mean MIC (% *v*/*v*).

Statistical analysis confirmed an inverse correlation between linalool content in the essential oil and the mean MIC values, indicating a link between higher linalool content and stronger antibacterial activity of the EO. The opposite relationship was observed for linalyl acetate (Figure 3).

**Figure 4:**
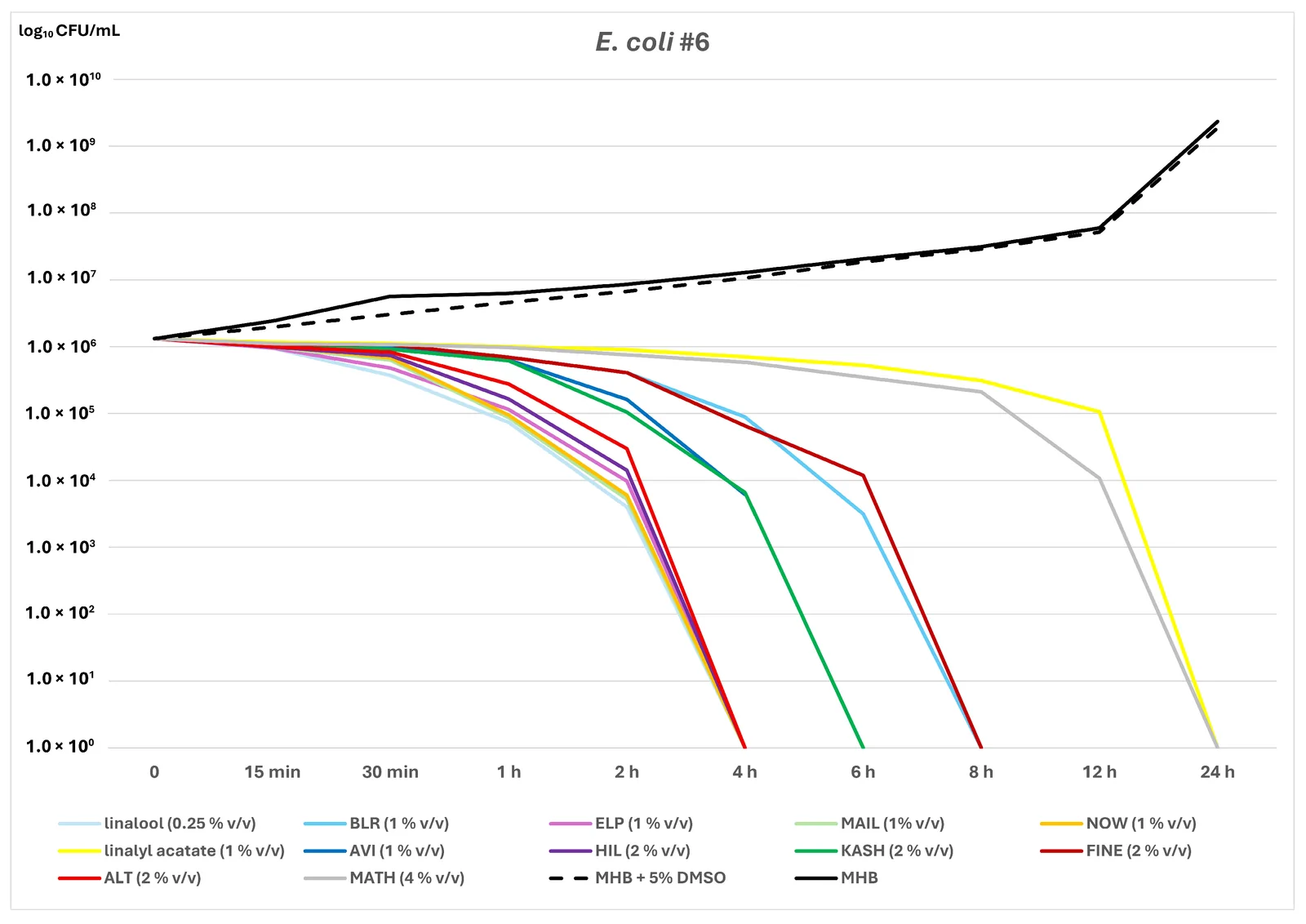
In vitro time–kill curves of *Escherichia coli* (strain number 6) exposed to various concentrations of lavender essential oils and their two main components (linalool and linalyl acetate). Bacterial suspension was incubated with the agent at the minimum inhibitory concentration (MIC) over 24 h. For each antimicrobial agent, the individual MIC values are listed in parentheses. Viable cell counts (*Y*-axis; log_10_ CFU/mL) were determined by serial dilution and plating at the specified point in time (*X*-axis). Data points represent the mean of *n* = 3 independent experiments. The solid multicolor lines indicate a reduction (99.9% killing) relative to the initial inoculum, defining bactericidal activity. Growth control without the antimicrobial agent is shown by the solid and dashed black lines.

The time–kill assay was performed to determine the time required to achieve complete eradication of *E. coli*, using strain 6 as a representative MDR strain and the antibiotic-susceptible reference *E. coli* strain ATCC 25922. All compounds were tested at concentrations corresponding to 1× MIC. Linalool and the ELP, MAIL, HIL, ALT, and NOW EOs required 4 h to completely eradicate the antibiotic-resistant strain 6 (Figure 4). A similar effect was observed after 6 h of incubation for the AVI and KASH EOs. Surprisingly, BLR EO required as long as 8 h to completely eliminate the antibiotic-resistant strain, despite showing the second-highest antibacterial activity after pure linalool in the MIC assay. FINE EO also required 8 h, although it was tested at a 2-fold higher concentration compared to BLR. Linalyl acetate and MATH EO, which was characterized by a high content (39.5%) of this compound, required as long as 24 h of incubation to completely eradicate strain 6.

**Figure 5:**
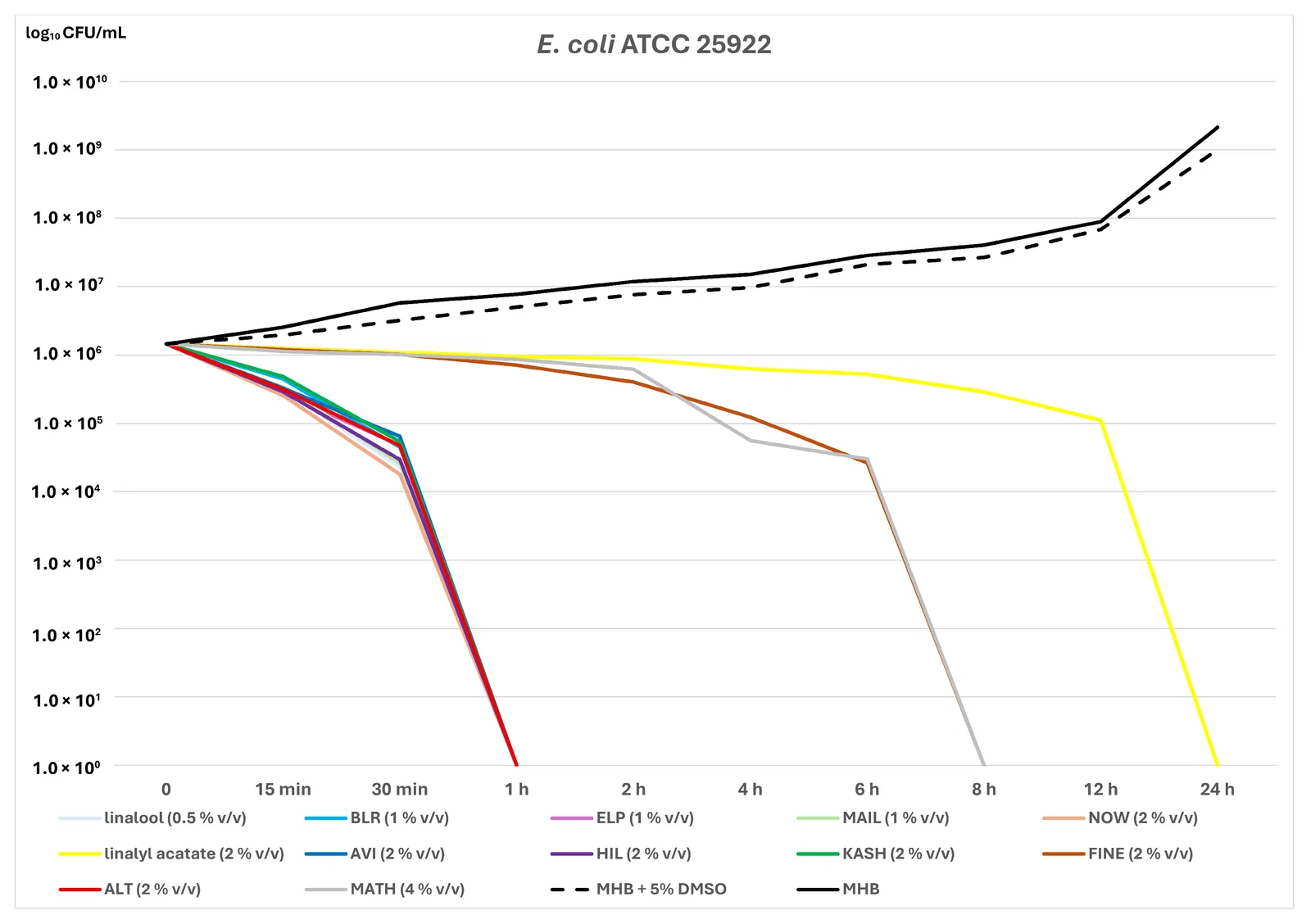
In vitro time–kill curves of *Escherichia coli* (ATCC 25922 reference strain) exposed to various concentrations of lavender essential oils and their two main components (linalool and linalyl acetate). Bacterial suspension was incubated with the agent at the minimum inhibitory concentration (MIC) over 24 h. For each antimicrobial agent, the individual MIC values are listed in parentheses. Viable cell counts (*Y*-axis; log_10_ CFU/mL) were determined by serial dilution and plating at the specified point in time (*X*-axis). Data points represent the mean of *n* = 3 independent experiments. The solid multicolor lines indicate a reduction (99.9% killing) relative to the initial inoculum, defining bactericidal activity. Growth control without the antimicrobial agent is shown by the solid and dashed black lines.

More consistent results were obtained for the reference *E. coli* strain ATCC 25922 (Figure 5). All essential oils containing ≥25% linalool required only 1 h to completely eradicate this strain. It should be noted, however, that these EOs were frequently tested at concentrations 2-fold higher than those used against *E. coli* strain 6, which may have contributed to the faster rate of bacterial eradication. The two EOs with the lowest linalool content (≤20%), i.e., FINE and MATH, required as long as 8 h to achieve the same effect. Again, pure linalyl acetate completely eradicated the reference strain only after 24 h of incubation.

## 3. Discussion

The chemical composition of EOs is highly variable, and their biological activity is largely determined by the dominant constituent [24]. Individual compounds, as well as their potential synergistic or antagonistic interactions, affect EO properties, which may contain mixtures of mono-, sesqui-, and diterpenes, as well as phenylpropanoids [15]. The approval of EOs for medical and veterinary applications requires strict control of their quality and composition, including analysis using standardized qualitative and quantitative methods [25].

In our microbiological analyses, linalool, one of the main components of lavender EOs, showed the highest antibacterial activity against the *E. coli* strains tested, with typical MIC values of 0.25% *v*/*v*, and sometimes as low as 0.125% *v*/*v*. Pure linalyl acetate was, on average, 5-fold less active than linalool (most common MIC: 1% *v*/*v*), which is consistent with previous reports suggesting that linalool often shows stronger antibacterial activity than its ester [26,27]. Nevertheless, the possible synergism of these two main components of lavender EO was also reported [28].

The antibacterial activity of pure linalool against an *E. coli* strain isolated from bovine uterine mucus and the reference *E. coli* strain ATCC 25922 was previously evaluated by Wang et al. [29]. The MIC and minimum bactericidal concentration (MBC) of linalool against these *E. coli* strains were 2 and 4 μL/mL, corresponding to approximately 0.173 and 0.35% *v*/*v*, respectively, assuming a mean linalool density of 0.865 g/cm^3^. These results are highly consistent with our findings (mean MIC, 0.31% *v*/*v*). Linalool is strongly lipophilic, allowing it to readily interact with the lipid bilayer of *E. coli*. This interaction alters membrane permeability, disrupts its potential, and ultimately causes irreversible damage, resulting in the leakage of cytoplasmic contents and bacterial cell death [30]. Linalool at concentrations of 2–8 μL/mL inhibited *E. coli* biofilm formation by 90–95% after 12 h of incubation. Moreover, this compound disrupted flagellar gene expression by downregulating fliA and motA and upregulating fliG and fliM, thereby impairing both the swarming and swimming motility of *E. coli* [29]. Gao et al. [31] demonstrated that linalool significantly affected *E. coli* lipopolysaccharide (LPS) synthesis and modification, inhibited ribosome biogenesis, and consequently, protein synthesis, and thus altered the expression of numerous proteins involved in the uptake of essential nutrients. Molecular docking studies have demonstrated that linalool has a high binding affinity for the target tripeptide glutathione [27], which protects bacteria against oxidative stress, osmotic shock, low pH, toxins, and heavy metals [32].

In our studies, we observed significant differences in the antibacterial activity of EOs obtained from various *L. angustifolia* varieties grown in different regions (e.g., ‘Blue River’ and ‘Ellagance Purple’ from Poland, ‘Kashmir’ from India, ‘Maillette’, ‘Matherone’, and ‘Fine’ from France, and ‘Highland’ from Greece). These can be attributed to differences in their chemical composition, particularly the proportions of linalool and linalyl acetate, as well as the presence of other, less abundant constituents that may act synergistically or additively. The analysis of the composition of the most active EOs against the tested *E. coli* strains showed that MAIL and ELP contained the highest levels of linalool (35.95% and 33.30%, respectively), consistent with their antibacterial activity (Figure 2 and Figure 3). It should be noted, however, that BLR, which demonstrated the highest antibacterial activity of all the EOs tested, contained a moderate level of linalool (24.63%), suggesting that it is not the only constituent that plays an important role in the antibacterial activity of lavender EOs. However, the slow inhibition of growth and delayed killing of *E. coli* isolate 6 by BLR (8 h), similar to that observed for the EOs with a high linalyl acetate and low linalool content (i.e., FINE and MATH), indicates that linalool plays a significant role in the rate of eradication of the planktonic form of this bacterium. A possible explanation for the high activity of this EO, as well as that of the other non-commercial EO, i.e., ELP, is the synergistic interaction of linalool and/or linalyl acetate with other constituents that are characteristic or unique components of these EOs. Composition analysis of the most active EO (BLR) showed that it contained the highest level of borneol (4.45%), which was also present at a relatively high level (3.60%) in ELP. Borneol is known for its antibacterial activity and has long been used in traditional Chinese and Indian medicine to treat gastrointestinal disorders [33]. The high antibacterial activity of borneol against *E. coli* has been confirmed in microbiological studies, in which an MIC of 512 μg/mL was reported for the reference *E. coli* strain ATCC 25922 [34]. Since the least active EOs contained significantly lower amounts of borneol (MATH, 0.51%; ALT, 0.56%), this may suggest that borneol contributes to the observed antibacterial activity. The BLR EO also contained a relatively high content of α-terpineol (4.99%), which is known for its strong antibacterial activity against *E. coli*. According to the literature, α-terpineol can induce morphological changes in *E. coli*, including reduced cell size, cell wall and membrane disruption, shrinkage of the cytoplasm, and loss of the nucleoid region at an MIC of 0.78 µL/mL [35]. Moreover, BLR was the only EO containing cryptone (1.84%), whose presence has also been associated with antibacterial activity in the literature [36]. It also contained the highest level of α-santalene (2.64%), which was detected in only one other EO (AVI), but at a substantially lower level (0.90%). Although no studies have reported a significant antibacterial effect of α-santalene against *E. coli*, it may act synergistically with other constituents, contributing to the overall antibacterial activity, as suggested for other minor components of the most active EOs. Further studies are needed to evaluate the antibacterial activity of these minor EO constituents and their interactions with the main components.

Variable antibacterial activity of lavender EOs against *E. coli* has also been reported by other authors. Studies have confirmed that lavender chemotype, determined by the dominant monoterpenes and their esters, directly affects its biological properties [28]. In the study by Stamova et al. [18], the highest susceptibility to lavender EO obtained from the variety ‘Jubileina’ (chemotype: 30% linalool and 24.3% linalyl acetate) was observed against the reference *E. coli* strain ATCC 25922, with both the MIC and MBC equal to 0.31% *v*/*v*. However, time–kill analysis showed that the number of viable cells after 24 h of incubation at this EO concentration was reduced by only 1.5 log_10_ CFU/mL. The bacterial count fell below the detection limit (<1 log10 CFU/mL) within 6 h only at the highest concentration tested (4 × MIC = 1.24% *v*/*v*), indicating near-complete eradication of the bacterial population in culture. This is consistent with our findings, in which the four most active EOs with a similar chemotype (BLR, ELP, MAIL, NOW) and MIC values ranging from 0.85 to 1.18% *v*/*v* completely eradicated the reference *E. coli* strain ATCC 25922 within 1 h. Moreover, molecular docking analysis has shown that both linalool and linalyl acetate bind to the NADH-binding pocket of the *E. coli* FabI protein, a key enzyme involved in bacterial fatty acid biosynthesis. Denkova et al. [37] also investigated the antibacterial activity of a lavender EO (chemotype: 42% linalyl acetate and 35.4% linalool) against the reference *E. coli* strain ATCC 25922. These authors reported a very low MIC of 500 µg/mL (0.06% *v*/*v*). However, their findings were not confirmed using other methods for assessing bacterial viability, such as MBC determination or time–kill analysis. In another study, a commercial lavender EO (chemotype: 31.74% linalyl acetate and 27.62% linalool) reached MIC values of 0.5% *v*/*v* against antibiotic-susceptible strains and 1% *v*/*v* against multidrug-resistant strains [38], which is consistent with our findings. Moreover, this EO showed clear synergistic activity with enrofloxacin. For the multidrug-resistant strain, a combination of lavender EO (1/4 × MIC = 0.25% *v*/*v*) and enrofloxacin (1/8 × MIC) was already effective after 4 h of incubation. For the reference *E. coli* strain ATCC 25922, enrofloxacin at 1/2 × MIC combined with lavender EO at 1/8 × MIC showed the same killing time and a similar killing profile. However, it remains unclear which of the two major constituents was responsible for this activity. Walasek-Janusz et al. [39] evaluated EOs obtained from two lavender varieties grown in eastern Poland: ‘Hidcote Blue Strain’ (chemotype: 16.9% linalyl acetate and 15.1% linalool) and ‘Hidcote Blue’ (chemotype: 34.2% linalyl acetate and 21.8% linalool). They reported identical MIC and MBC values of 10 mg/mL for the reference *E. coli* strain ATCC 25922, corresponding to an EO concentration of approximately 1% *v*/*v*, which is consistent with our findings. EOs obtained from two Romanian lavender varieties, ‘Vera’ (chemotype: 29.4% linalool and 26.8% linalyl acetate) and ‘Sevtopolis’ (chemotype: 35.6% linalool and 6% linalyl acetate), effectively eliminated the reference *E. coli* strain ATCC 25922 at a concentration of 4 mg/mL (approximately 0.4% *v*/*v*) [40]. Dinçoğlu and Çalışkan [41] investigated the antibacterial activity of an EO (chemotype: 37% linalool and 28.7% linalyl acetate) obtained from lavender grown in Burdur, Turkey. The antibacterial activity of the oil was evaluated against the *E. coli* O157:H7 serotype, a Shiga toxin-producing *E. coli* (STEC) strain and one of the most important foodborne pathogens, primarily associated with meat and its products. The MIC for *E. coli* in these studies was determined to be 6.4 μL/mL (approximately 0.64% *v*/*v*), while the MBC was 12.8 μL/mL (approximately 1.28% *v*/*v*). However, a reduction of approximately 3 log in viable *E. coli* O157:H7 cells was observed only when the EO was applied at twice the MIC (equivalent to the MBC), corresponding to approximately 1.28% *v*/*v*. This is in agreement with our findings and those reported by Djenane et al. [42] for lavender EO (chemotype: 22.35% linalool and 21.80% linalyl acetate) obtained from plants cultivated in the Kabylia region of Algeria. Abdulkarim and Issa [43] evaluated the antimicrobial activity of an Iraqi lavender EO (chemotype unknown) against an *E. coli* isolate obtained from clinical cases of pneumonia in sheep. The authors reported an MIC similar to that determined in our study (12 μL/mL, approximately 1.2% *v*/*v*). In time–kill assays performed at the MBC (15 μL/mL, approximately 1.5% *v*/*v*), the EO required more than 4 h to completely eliminate the bacterium.

Another study evaluated a lavender EO with a high linalool content (chemotype: 47.55% linalool and only 3.75% linalyl acetate). It showed stronger antibacterial activity (MIC and MBC: 0.1–0.19% *v*/*v*) against selected bacterial strains (*E. coli* ATCC 25922 and *E. coli* ESBL 4493) compared to basil EO [44]. The *E. coli* O157:H7 ATCC 32158 strain was also highly susceptible (MIC: 2000 µg/mL, approximately 0.2% *v*/*v*) to both linalool and lavender EO (chemotype: 53.5% linalool and 4.2% linalyl acetate) [45]. Similarly, Thielmann et al. [46] reported that the reference *E. coli* strain DSM 1103 was susceptible to three lavender EOs obtained from different manufacturers (chemotypes unknown), with MIC values ranging from 800 to 1600 μg/mL (0.08–0.16% *v*/*v*). However, the authors did not perform additional analyses of cell viability at these low concentrations. A commercial lavender EO with a more balanced chemotype, Hanus (Nitra, Slovakia), obtained from a Spanish lavender cultivar (chemotype: 34.3% linalool and 37.4% linalyl acetate), showed high antibacterial activity against the *E. coli* reference strain CCM 3953, with an MIC of 1 mg/mL (approximately 0.1% *v*/*v*) [47]. In contrast, Puvača et al. [48] analyzed a commercial lavender EO from Novi Sad, Serbia, with an atypical chemotype (10.7% linalool and 9.6% linalyl acetate). Its antibacterial activity against a clinical human *E. coli* isolate and the reference *E. coli* strain ATCC 25922 strain was very low (>5% *v*/*v*). This may indicate that the linalool/linalyl acetate content plays an important role in the antibacterial activity of lavender EOs.

Overall, the MIC values of lavender EOs vary depending on their chemotype, geographical origin, and numerous other factors. However, reported values generally range between 100 µg/mL (approximately 0.01% *v*/*v*) and 10 mg/mL (approximately 1% *v*/*v*). These values are not necessarily indicative of high antibacterial activity. Strict criteria for evaluating the antimicrobial activity of EOs have been proposed by Saraiva et al. [49]. According to these authors, MIC values below 100 µg/mL (approximately 0.01% *v*/*v*) indicate high antimicrobial activity. An MIC between 100 and 500 µg/mL (approximately 0.01–0.05% *v*/*v*) is considered to indicate moderate antimicrobial activity. MIC values between 500 and 1000 µg/mL (approximately 0.05–0.1% *v*/*v*) indicate low antimicrobial activity, whereas EOs with MIC values above 2000 µg/mL (approximately 0.2% *v*/*v*) are considered inactive. Thus, the antimicrobial activity of lavender EO can be considered, at best, moderate. Its potential use on farms is further limited by its cost, as well as its taste and aroma. Therefore, efforts should be directed toward reducing the effective EO concentration below 100 µg/mL. A potential approach is to investigate interactions between EOs and other antimicrobial agents, for example, using the checkerboard assay. Initial studies have provided promising results, including a strong synergistic interaction between lavender EO and enrofloxacin against APEC strains [38].

## 4. Materials and Methods

### 4.1. Essential Oils and Their Major Constituents

Ten various *L. angustifolia* EOs of different origins were used in this study (see: Table 1). Two EOs were isolated from the varieties ‘Blue River’ (BLR) and ‘Ellagance Purple’ (ELP). The plant material was obtained from the Experimental Station of the Department of Horticulture at the West Pomeranian University of Technology in Szczecin (Poland). The EOs were isolated according to the methodology described in our previous study [50]. The remaining eight EOs were commercially available. Three of the commercial EOs were obtained from the French lavender varieties ‘Lavender Maillette’ (MAIL), ‘Lavender Matherone’ (MATH) (Oshadhi™, Fairfield, IA, USA), and ‘Lavender Fine’ (FINE) (Fleurance Nature™, Fleurance, France). One EO (NOW) was a blend of oils from several French lavender varieties (Now^®^ Foods, Bloomingdale, IL, USA). Two EOs originated from Bulgarian lavender: ALT, obtained from plants cultivated in the Rose Valley region (Alteya Organics™, Yagoda, Bulgaria), and AVI, supplied by a Polish manufacturer (Avicenna Oil, Wrocław, Poland). The study also included an EO from the variety ‘Highland Lavender’ (HIL), obtained from high-altitude plantations in Greece (Plant Therapy™, Twin Falls, ID, USA), and an EO from the variety ‘Kashmir Lavender’ (KASH), cultivated in India (Oshadhi™, Fairfield, IA, USA). In addition, the two major compounds determining the chemotype of lavender EO and potentially responsible for its antimicrobial activity, linalool (>96% purity) and linalyl acetate (>95% purity) (TCI, Tokyo Chemical Industry, Tokyo, Japan), were tested directly in the microbiological assays.

### 4.2. GC–MS Analysis of the Chemical Composition of Essential Oils

The chemical composition of the EOs was analyzed by the GC-MS method using a 6890N Gas Chromatograph equipped with a 5973N Mass Selective Detector and a 7683 Series Autosampler (Agilent Technologies, Santa Clara, CA, USA). Before analysis, 20 μL of each EO was diluted in 1 mL of acetone (analytical grade) in a 1.5-mL chromatographic vial. Next, 3 μL of the resulting solution was injected in triplicate onto an HP-5MS column (30 m × 0.25 mm i.d., 0.25 μm film thickness) using a split ratio of 10:1. Helium (5.0) was used as the carrier gas at a flow rate of 1.2 mL/min. The oven temperature was programmed from 50 °C to 290 °C at a rate of 4 °C/min. The temperatures of the injector, ion source, and quadrupole were set at 250 °C, 230 °C, and 150 °C, respectively. Mass spectra of the separated compounds were acquired by electron impact ionization (70 eV) over an *m*/*z* range of 20–500. The compounds were identified by matching their mass spectra with the NIST 04 library and by comparing their chromatographic data with an in-house database of reference standards. Compound identification was further confirmed by calculating the experimental linear retention indices (RI_exp_) and comparing them with the published values (RI_lit_) [51]. For this purpose, the retention times of the *n*-alkane standard (C7–C30, Supelco, Bellefonte, PA, USA) were determined under the same chromatographic conditions. The experimental retention indices were subsequently calculated using the following equation:

RI_exp_ = 100n + 100 (RT_x_ − RT_n_)/(RT_n + 1_ − RT_n_) (1)

where:

RI_exp_—linear retention index determined experimentally,

RT_x_—retention time of analyzed compound [min],

RT_n_—retention time of the n-alkane eluting immediately before the analyzed compound (min),

RT_n + 1_—retention time of the n-alkane eluting immediately after the analyzed compound (min).

The relative percentage contents of individual EO constituents was determined by the area normalization method, based on the ratio of the peak area of each compound to the total peak area of all compounds detected in the sample. The obtained values are presented in Table 2. The retention indices (RI_exp_ and RI_lit_) as well as examples of the chromatograms and mass spectra are provided in the Supplementary Materials (Table S1, Figures S1–S12).

### 4.3. Bacterial Strains

The antimicrobial activity of lavender EOs was evaluated against 19 field strains of the Gram-negative bacterium *E. coli*, isolated in 2021–2022 from clinical cases of omphalitis and yolk sac infection in one-day-old broiler chicks at a veterinary diagnostic laboratory in Poland. The strains were preserved using ViaBank™ bacterial storage systems (MVE, Medical Wire & Equipment, Potley Lane, Corsham, UK) and stored at temperatures below −20 °C until analysis. The reference *E. coli* strain ATCC 25922 (WDCM 00013; KWIK-STIK™ Plus, Microbiologics, St. Cloud, MN, USA) was also included in the study. Antimicrobial resistance data obtained from the official laboratory reports are provided in our other article [52] (Supplementary Materials, Table S1). Strains 1–10 were classified as multidrug-resistant (MDR), defined as non-susceptible to at least three different classes of antibiotics. Strains 11–19 and the reference *E. coli* strain ATCC 25922 were susceptible to conventional antimicrobial agents used in poultry medicine. Twenty-four hours before inoculation of the test plates, each strain was revived on Columbia agar supplemented with 5% sheep blood (Graso, Starogard Gdański, Poland) and incubated at 37 ± 1 °C.

### 4.4. Antibacterial Activity Assay

Each EO, as well as linalool and linalyl acetate, was initially diluted in dimethyl sulfoxide (DMSO) (Warchem, Warsaw, Poland) to a working concentration of 80% (*v*/*v*). Antibacterial activity was determined by the MIC method using sterile 96-well microplates with lids (Wuxi Nest Biotechnology, Wuxi, China). Cation-adjusted Mueller–Hinton broth (CAMHB) (GRASO, Gdańsk, Poland) was used as the test medium. The wells in the first row were filled with 180 μL of CAMHB and 20 μL of the working solution of each EO, linalool, or linalyl acetate, resulting in an initial test concentration of 8% (*v*/*v*). The contents were thoroughly mixed using a multichannel pipette, and 100 μL of the resulting mixture was transferred to the next row containing 100 μL of CAMHB. The contents were then mixed again. This procedure was repeated for the subsequent rows, resulting in a twofold dilution series ranging from 8% to 0.125% (*v*/*v*). The last row was the positive growth control that contained 100 μL of MHB without the antimicrobial agent. The microplate was inoculated by adding 10 μL of the selected *E. coli* strain, previously adjusted to 0.5 McFarland and then diluted 10-fold to obtain the working bacterial suspension. As a result, the final bacterial concentration in each well was approximately 1.0 × 10^6^ CFU/mL. The inoculated microplates were incubated for 18 h at 36 ± 1 °C.

### 4.5. MIC Determination and Visualization

Lavender EO at concentrations ≥0.5% (*v*/*v*) may cause natural turbidity of CAMHB, and prolonged incubation may result in precipitate formation (authors’ observations). As a result, MIC determination may be difficult, or in some cases, impossible, leading to false results. Therefore, after 18 h of incubation, 20 μL of sterile 0.01% resazurin solution (POL-AURA, Olsztyn, Poland) was added to each well, followed by a further 6 h of incubation. Resazurin is a dark blue dye that is reduced to pink in the presence of viable cells, thereby indicating EO concentrations at which bacterial growth still occurs. The MIC was defined as the lowest EO concentration at which the color of the bacterial culture remained unchanged (blue) after a total incubation time of 24 h. An example of a plate for MIC determination after incubation and the addition of resazurin is shown in the Supplementary Materials (Figure S13).

### 4.6. Time–Kill Analysis

The rate of antibacterial activity at the effective concentrations of the EOs, linalool, and linalyl acetate was evaluated based on the MIC values determined for each compound. Pure CAMHB and CAMHB supplemented with 5% DMSO [53] were also included as controls. For this purpose, one strain was selected from each of the two predefined antibiotic susceptibility categories (resistant and susceptible). Each tube contained a final volume of 10 mL of CAMHB supplemented with the appropriate concentration of the test antimicrobial agent, and as in the microplate assay, an initial bacterial concentration of approximately 1.0 × 10^6^ CFU/mL. Directly after each specified incubation period, viable cell counts were determined from a 1-mL sample collected at 0 min (initial inoculum control) and after 15 min, 30 min, 1 h, 2 h, 4 h, 6 h, 8 h, 12 h, and 24 h. The number of viable *E. coli* cells was determined in accordance with ISO 4833-1:2013 [54]. Briefly, immediately after collection, each 1-mL sample was subjected to 10-fold serial dilutions in 0.9% saline (Ecotainer^®^, B. Braun Medical AG, Sempach, Switzerland) on ice. Subsequently, 1 mL of each dilution was transferred to a sterile Petri dish, overlaid with nutrient agar (Graso, Starogard Gdański, Poland), and gently mixed. After the agar had completely solidified, the plates were inverted and incubated at 30 °C for 72 h. Colony counting and calculation were performed in accordance with ISO 7218 [55].

### 4.7. Statistical Analysis

All analyses were performed in triplicate, and the results are presented as mean ± standard deviation (SD). Classical cluster analysis using Ward’s linkage method and Euclidean distance, as well as correlations between the contents of the main EO constituents and their antibacterial activity were performed using PQStat v.1.8.4. Both linear and rank correlation analyses were performed. Results were considered statistically significant at *p* < 0.05.

## 5. Conclusions

The results of the present study confirm that EOs from *Lavandula angustifolia* and their major constituents, particularly linalool, possess significant antibacterial activity against various *E. coli* strains, including multidrug-resistant isolates, which represent a major challenge in commercial poultry production. Variation in lavender chemotypes significantly affects their antibacterial activity, indicating the need for the standardization and chemical characterization of EOs intended for medical and veterinary applications. Further research in this area, including studies on the potential use of EOs to enhance the activity of conventional antimicrobial agents, may contribute to the development of new and effective strategies for controlling antimicrobial-resistant pathogens within the One Health initiative.

## Figures and Tables

**Table 1:** Characteristics of the tested *L. angustifolia* essential oils and their code designations.

Essential Oil	Abbreviation									
	BLR	ELP	AVI	FINE	MAIL	MATH	ALT	HIL	KASH	NOW
Variety	Blue River	Ellagance Purple	unknown	Fine	Maillette	Matherone	unknown	Highland	Kashmir	unknown (mixed)
Country of lavender’s origin	Poland	Poland	Bulgaria	France	France	France	Bulgaria	Greece	India	France
Source or manufacturer	ZUT ^1^	ZUT ^1^	Avicenna Oil	Fleurance Nature	Oshadhi	Oshadhi	Alteya Organics	Plant Therapy	Oshadhi	Now Foods
Country of manufacture	Poland	Poland	Poland	France	USA	USA	Bulgaria	USA	USA	USA

^1^ ZUT—West Pomeranian University of Technology in Szczecin [17].

**Table 2:** GC-MS analysis of the chemical composition of *Lavandula angustifolia* essential oils.

No.	Compound	*Lavandula angustifolia* EO									
		BLR	ELP	AVI	FINE	MAIL	MATH	ALT	HIL	KASH	NOW
		Relative Content ± SD ^1^ [%]									
**1**	α-Pinene	-	-	-	0.19 ± 0.06	-	0.17 ± 0.05	0.18 ± 0.01	0.41 ± 0.08	-	0.46 ± 0.07
**2**	Camphene	-	-	-	0.22 ± 0.07	0.26 ± 0.03	0.11 ± 0.02	-	0.14 ± 0.02	0.32 ± 0.06	0.22 ± 0.03
**3**	β-Pinene	-	0.69 ± 0.08	-	-	-	-	-	-	-	-
**4**	3-Octanone	0.65 ± 0.01	-	-	-	1.67 ± 0.24	-	1.27 ± 0.22	-	-	1.13 ± 0.16
**5**	β-Myrcene	-	-	-	-	-	0.11 ± 0.04	0.49 ± 0.01	-	0.27 ± 0.19	-
**6**	Hexyl acetate	0.46 ± 0.05	-	-	-	-	-	0.70 ± 0.12	-	-	-
**7**	1,8-Cineole	1.60 ± 0.03	1.83 ± 0.05	0.53 ± 0.02	1.98 ± 0.12	-	1.34 ± 0.05	1.83 ± 0.09	0.60 ± 0.10	1.22 ± 0.43	2.70 ± 0.10
**8**	*cis*-β-Ocimene	-	-	-	3.92 ± 0.16	1.39 ± 0.04	4.81 ± 0.61	2.80 ± 0.13	7.54 ± 0.48	1.11 ± 0.15	2.21 ± 0.16
**9**	*trans*-β-Ocimene	-	0.96 ± 0.04	-	3.94 ± 0.15	0.92 ± 0.08	8.57 ± 0.41	2.62 ± 0.13	3.24 ± 0.43	1.44 ± 0.25	0.90 ± 0.05
**10**	*cis*-Linalool oxide	2.79 ± 0.08	6.13 ± 0.06	0.96 ± 0.06	-	-	-	-	-	-	-
**11**	*trans*-Linalool oxide	2.25 ± 0.07	5.23 ± 0.08	0.67 ± 0.04	-	-	-	-	-	-	-
**12**	**Linalool**	**24.63 ± 0.42**	**33.30 ± 0.39**	**25.86 ± 0.60**	**20.36 ± 0.62**	**35.95 ± 0.79**	**16.00 ± 0.21**	**26.77 ± 0.14**	**25.03 ± 0.53**	**28.15 ± 0.96**	**29.32 ± 0.44**
**13**	1-Octen-3-yl acetate	1.36 ± 0.04	0.53 ± 0.01	1.05 ± 0.04	1.62 ± 0.05	1.15 ± 0.02	1.64 ± 0.11	1.36 ± 0.04	2.29 ± 0.43	1.45 ± 0.44	1.70 ± 0.04
**14**	Pinocarveol	-	0.76 ± 0.07	-	-	-	--	-	-	-	-
**15**	Camphor	0.65 ± 0.03	0.90 ± 0.03	0.36 ± 0.01	0.45 ± 0.02	0.96 ± 0.08	0.24 ± 0.01	0.23 ± 0.01	0.41 ± 0.09	0.53 ± 0.05	0.48 ± 0.03
**16**	Borneol	4.45 ± 0.08	3.60 ± 0.09	2.20 ± 0.04	1.15 ± 0.02	2.54 ± 0.21	0.51 ± 0.03	0.56 ± 0.03	0.44 ± 0.02	2.25 ± 0.20	0.99 ± 0.03
**17**	Lavandulol	-	-	0.39 ± 0.02	1.08 ± 0.09	1.56 ± 0.78	0.58 ± 0.21	0.83 ± 0.13	-	-	1.30 ± 0.14
**18**	Terpinen-4-ol	3.34 ± 0.04	1.72 ± 0.04	2.53 ± 0.05	3.70 ± 0.02	3.80 ± 0.04	2.70 ± 0.03	4.09 ± 0.03	7.24 ± 0.25	1.30 ± 0.10	7.46 ± 0.18
**19**	Cryptone	1.84 ± 0.02	-	-	-	-	-	-	-	-	-
**20**	α-Terpineol	4.99 ± 0.02	2.65 ± 0.07	7.23 ± 0.27	1.67 ± 0.06	2.67 ± 0.12	-	2.32 ± 0.11	1.51 ± 0.19	2.34 ± 0.52	2.20 ± 0.07
**21**	**Linalyl acetate**	**25.15 ± 0.34**	**18.15 ± 0.77**	**32.78 ± 0.18**	**34.98 ± 0.91**	**33.25 ± 1.59**	**39.50 ± 0.77**	**30.96 ± 0.62**	**35.03 ± 0.54**	**43.61 ± 1.08**	**31.55 ± 0.39**
**22**	Bornyl acetate	0.54 ± 0.05	-	0.60 ± 0.02	-	0.38 ± 0.10	0.14 ± 0.06	-	0.30 ± 0.03	0.50 ± 0.12	0.22 ± 0.01
**23**	Lavandulyl acetate	7.50 ± 0.31	4.12 ± 0.09	7.87 ± 0.16	5.48 ± 0.16	2.48 ± 0.17	9.58 ± 0.09	5.09 ± 0.06	5.47 ± 0.39	3.99 ± 0.38	3.54 ± 0.10
**24**	Neryl acetate	1.45 ± 0.04	0.95 ± 0.08	1.91 ± 0.05	-	0.61 ± 0.10	-	-	-	0.50 ± 0.30	-
**25**	Geranyl acetate	2.69 ± 0.12	1.98 ± 0.08	3.94 ± 0.13	0.95 ± 0.01	1.01 ± 0.06	-	0.97 ± 0.05	-	1.20 ± 0.25	0.90 ± 0.12
**26**	β-Caryophyllene	-	2.06 ± 0.03	1.03 ± 0.02	9.17 ± 0.40	5.13 ± 0.19	8.15 ± 0.18	6.17 ± 0.05	5.27 ± 0.25	5.95 ± 0.23	5.25 ± 0.16
**27**	α-Santalene	2.64 ± 0.14	-	0.90 ± 0.02	-	-	-	-	-	-	-
**28**	*trans*-α-Bergamotene	-	-	0.16 ± 0.01	0.30 ± 0.04	0.23 ± 0.03	0.23 ± 0.06	0.24 ± 0.01	0.18 ± 0.04	0.18 ± 0.02	0.22 ± 0.01
**29**	α-Humulene	-	-	-	0.29 ± 0.03	-	0.21 ± 0.02	0.16 ± 0.02	-	0.18 ± 0.03	-
**30**	*trans*-β-Farnesene	0.65 ± 0.03	1.65 ± 0.04	0.50 ± 0.01	4.44 ± 0.37	1.66 ± 0.02	3.65 ± 0.03	7.93 ± 0.05	3.51 ± 0.10	1.40 ± 0.19	3.91 ± 0.09
**31**	Germacrene D	-	-	-	0.58 ± 0.10	-	0.74 ± 0.07	1.19 ± 0.04	0.34 ± 0.14	0.18 ± 0.03	-
**32**	γ-Cadinene	0.62 ± 0.04	0.59 ± 0.05	0.26 ± 0.07	0.29 ± 0.01	-	-	0.22 ± 0.01	-	0.16 ± 0.04	0.29 ± 0.02
**33**	Caryophyllene oxide	5.09 ± 0.17	5.13 ± 0.05	2.69 ± 0.20	2.10 ± 0.11	1.11 ± 0.15	0.71 ± 0.04	0.39 ± 0.01	0.37 ± 0.05	1.47 ± 0.18	2.54 ± 0.14
**34**	*epi*-Bicyclose-squiphellandrene	2.32 ± 0.08	1.18 ± 0.08	0.66 ± 0.05	0.26 ± 0.01	-	-	-	-	-	-

^1^ SD—standard deviation (*n* = 3). The main components and their contents are highlighted in bold.

**Table 3:** Antimicrobial activity of ten lavender essential oils and their two principal components against twenty *Escherichia coli* strains expressed as minimum inhibitory concentration (MIC, % *v*/*v*).

*E. coli* Strain		MIC ^1^ [% *v*/*v*]											
				*L. angustifolia* EOs and Their Main Constituents									
		Linalool	Linalyl Acetate	BLR	ELP	AVI	FINE	MAIL	MATH	ALT	HIL	KASH	NOW
MDR ^2^	1	0.5	2	1	1	2	2	1	2	2	2	2	1
	2	0.25	1	1	1	2	2	1	4	2	2	2	1
	3	0.25	4	1	2	2	4	1	8	4	2	2	1
	4	0.25	1	1	1	2	4	1	4	2	2	1	1
	5	0.25	0.5	0.5	0.5	1	1	0.5	2	1	1	1	0.5
	6	0.25	1	1	1	1	2	1	4	2	2	2	1
	7	0.125	1	1	1	1	2	1	4	2	1	1	1
	8	0.25	4	1	1	4	4	1	8	4	2	2	2
	9	0.5	4	1	1	2	8	1	8	4	2	2	1
	10	0.25	4	1	1	2	2	1	8	4	2	4	1
S ^3^	11	0.25	0.5	0.5	1	1	2	1	2	2	2	2	1
	12	0.125	0.5	0.5	0.5	1	1	1	2	2	1	1	1
	13	0.5	1	1	1	2	2	1	2	4	2	2	1
	14	0.25	1	0.5	1	1	2	1	2	2	1	2	1
	15	0.25	1	1	1	2	4	1	8	2	2	2	2
	16	0.25	1	0.5	1	1	2	1	4	4	2	2	1
	17	0.5	1	1	1	2	2	1	4	2	2	2	2
	18	0.25	1	0.5	1	1	1	1	2	1	1	1	1
	19	0.5	1	1	1	2	2	1	8	2	2	1	1
	ATCC ^4^	0.5	2	1	1	2	2	1	4	4	2	2	2
MIC range	MIN	0.125	0.5	0.5	0.5	1	1	0.5	2	1	1	1	0.5
	MAX	0.5	4	1	2	4	8	1	8	4	2	4	2
Mean MIC [% *v*/*v*]		0.31	1.63	0.85	1.00	1.70	2.55	0.98	4.50	2.60	1.75	1.80	1.18
The most frequent MICs	[% *v*/*v*]	0.25	1	1	1	2	2	1	4	2	2	2	1
	[% of strains]	60	55	70	85	55	60	95	35	55	75	65	75

^1^ MIC—minimal inhibitory concentration; ^2^ MDR—multidrug-resistant strain; ^3^ S—susceptible strain; ^4^ ATCC—*E. coli* ATCC 25922 reference strain.

## Data Availability

The original contributions presented in this study are included in the article/Supplementary Materials. Further inquiries can be directed to the corresponding author.

## Supplementary Materials

The following supporting information can be downloaded at https://www.mdpi.com/article/10.3390/molecules31193495/s1, Table S1: Retention indices of compounds identified in *L. angustifolia* essential oils, obtained experimentally by the GC-MS method and compared with the literature data; Figure S1: GC-MS chromatogram of essential oil from *L. angustifolia* variety ‘Blue River’ (BLR); Figure S2: GC-MS chromatogram of essential oil from *L. angustifolia* variety ‘Ellagance Purple’ (ELP); Figure S3: GC-MS chromatogram of essential oil from Bulgarian *L. angustifolia* produced by Avicenna (AVI); Figure S4: GC-MS chromatogram of essential oil from *L. angustifolia* variety ‘Fine’ (FINE); Figure S5: GC-MS chromatogram of essential oil from *L. angustifolia* variety ‘Maillette’ (MAIL); Figure S6: GC-MS chromatogram of essential oil from *L. angustifolia* variety ‘Matherone’ (MATH); Figure S7: GC-MS chromatogram of essential oil from Bulgarian *L. angustifolia* produced by Alteya Organics (ALT); Figure S8: GC-MS chromatogram of essential oil from *L. angustifolia* variety ‘Highland’ (HIL); Figure S9: GC-MS chromatogram of essential oil from *L. angustifolia* variety ‘Kashmir’ (KASH); Figure S10: GC-MS chromatogram of essential oil from French *L. angustifolia* varieties produced by Now Foods (NOW); Figure S11: GC-MS chromatogram of linalool (a) and its mass spectrum compared with the linalool standard mass spectrum from the NIST 04 library (b); Figure S12: GC-MS chromatogram of linalyl acetate (a) and its mass spectrum compared with the linalyl acetate standard mass spectrum from the NIST 04 library (b); Figure S13: Example of plate for MIC determination after 24 h of incubation and the addition of resazurin for the antibiotic-sensitive *E. coli* ATCC 25922 strain.

## Abbreviations

The following abbreviations are used in this manuscript:

APEC	Avian pathogenic *Escherichia coli*
EO	Essential oil
MIC	Minimum inhibitory concentration
