# Supplementary material for: Evaluation of Different Chemotypes of Lavandula angustifolia Essential Oils as Phytobiotics for the Control of Clinical Escherichia coli Strains Isolated from Commercial Poultry Production
Source: Molecules. 2026 Oct 1;31(19):3495. doi: 10.3390/molecules31193495 (PMC13666156; doi:10.3390/molecules31193495)
Supplement: Supplementary file 1 [file molecules-31-03495-s001.zip › molecules-4571885-supplementary.pdf]

Supplementary Materials

Table S1. Retention indices of compounds identified in *L. angustifolia* essential oils, obtained experimentally by GC-MS method and compared with literature data

No.	Compound	RI_{exp}[1]	RI_{lit}[2]
1	α-Pinene	931	936
2	Camphene	945	950
3	β-Pinene	973	977
4	3-Octanone	990	985
5	β-Myrcene	994	989
6	Hexyl acetate	1014	1010
7	1,8-Cineole	1030	1032
8	*cis*-β-Ocimene	1040	1038
9	*trans*-β-Ocimene	1050	1048
10	*cis*-Linalool oxide	1077	1075
11	*trans*-Linalool oxide	1089	1083
12	Linalool	1106	1099
13	1-Octen-3-yl acetate	1113	1110
14	Pinocarveol	1142	1140
15	Camphor	1144	1143
16	Borneol	1168	1166
17	Lavandulol	1171	1168
18	Terpinen-4-ol	1179	1177
19	Cryptone	1189	1187
20	α-Terpineol	1196	1190
21	Linalyl acetate	1258	1255
22	Bornyl acetate	1286	1284
23	Lavandulyl acetate	1293	1289
24	Neryl acetate	1368	1362
25	Geranyl acetate	1385	1380
26	β-Caryophyllene	1417	1419
27	α-Santalene	1421	1422
28	*trans*-α-Bergamotene	1435	1435
29	α-Humulene	1453	1453
30	*trans*-β-Farnesene	1458	1456
31	Germacrene D	1480	1481
32	γ-Cadinene	1515	1513
33	Caryophyllene oxide	1582	1581
34	*epi*-Bicyclosesquiphellandrene	1645	1642

[1] RI_{exp} - Linear retention index determined experimentally in relation to n-alkanes (C7-C30) on a HP5-MS column; [2] RI_{lit} - Reference linear retention index from the literature [51].

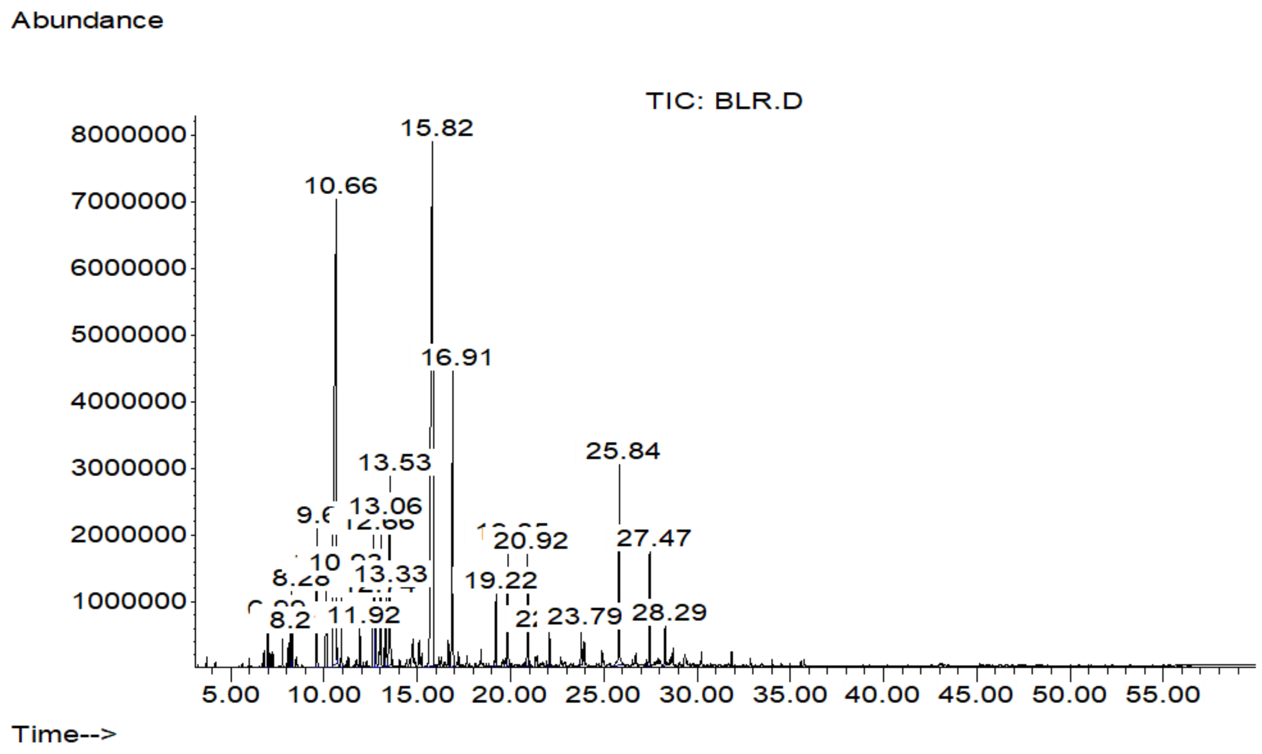

Figure S1. GC-MS chromatogram of essential oil from *L. angustifolia* variety ‘Blue River’ (BLR)

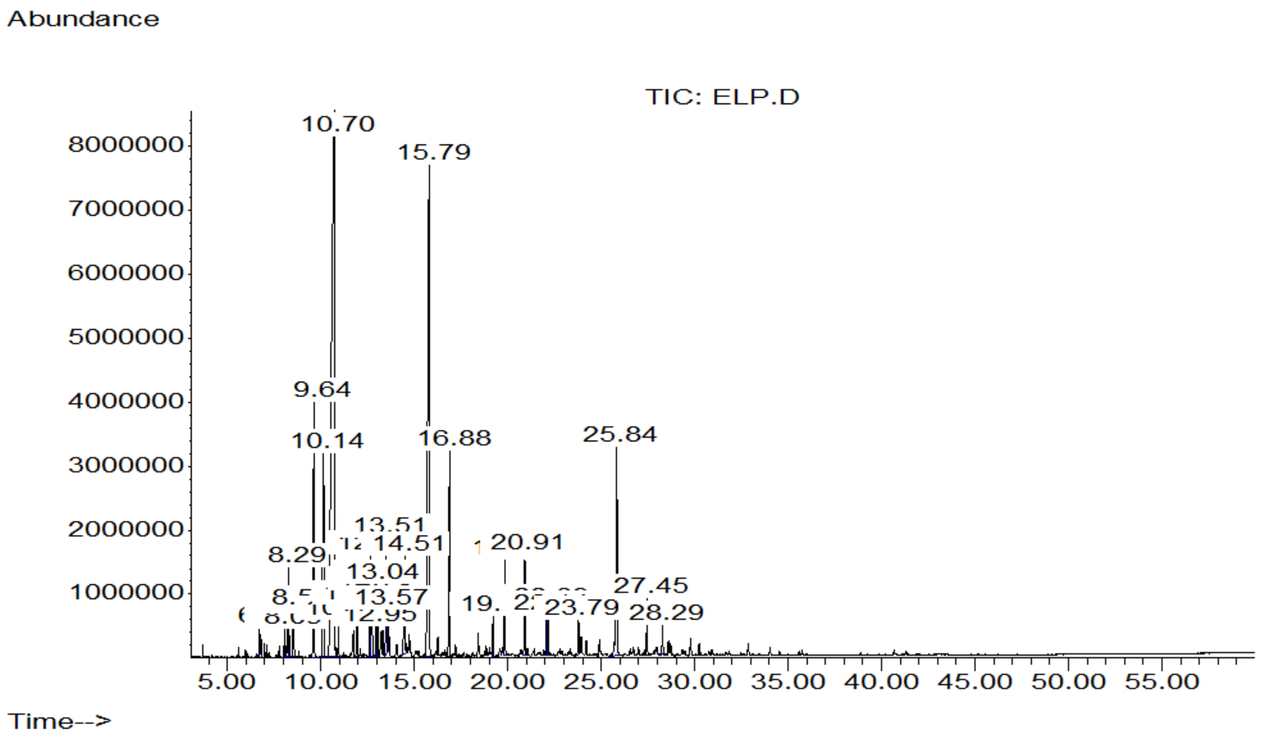

Figure S2. GC-MS chromatogram of essential oil from *L. angustifolia* variety ‘Ellagance Purple’ (ELP)

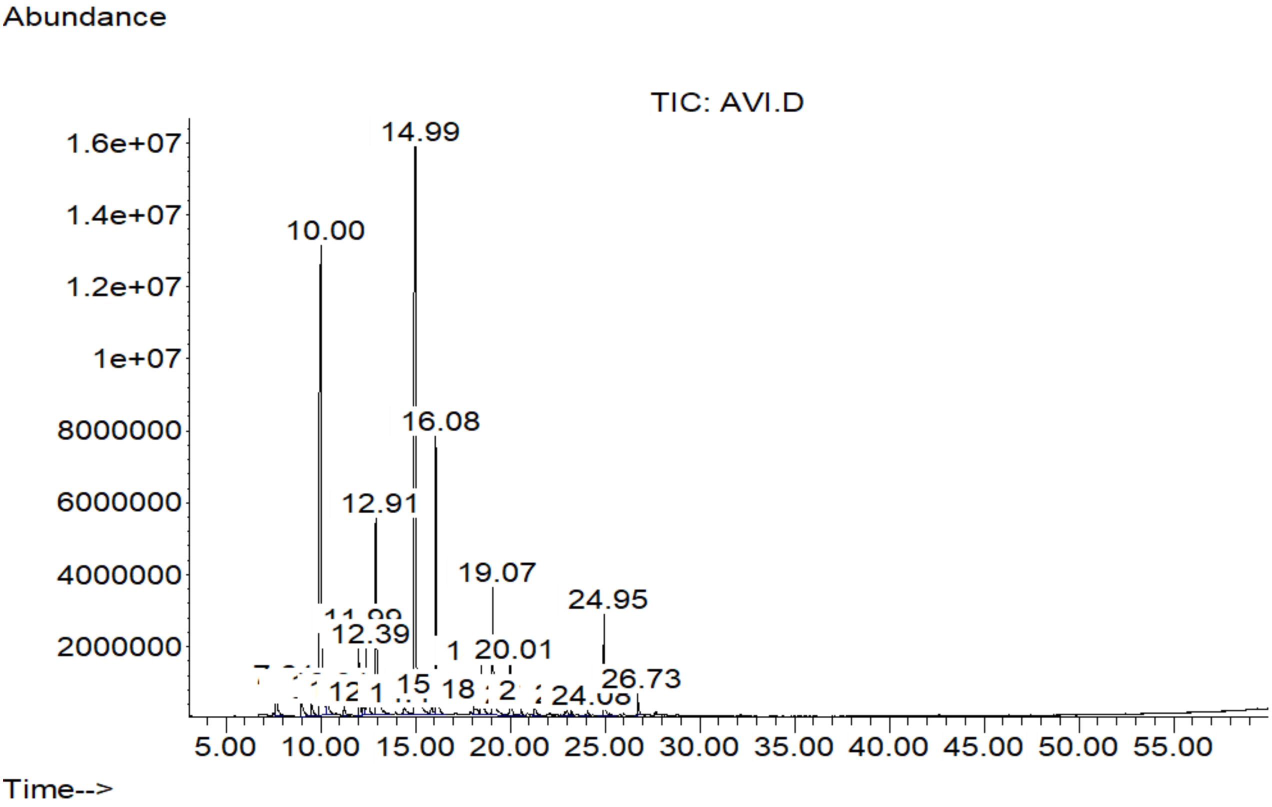

Figure S3. GC-MS chromatogram of essential oil from Bulgarian *L. angustifolia* produced by Avicenna (AVI)

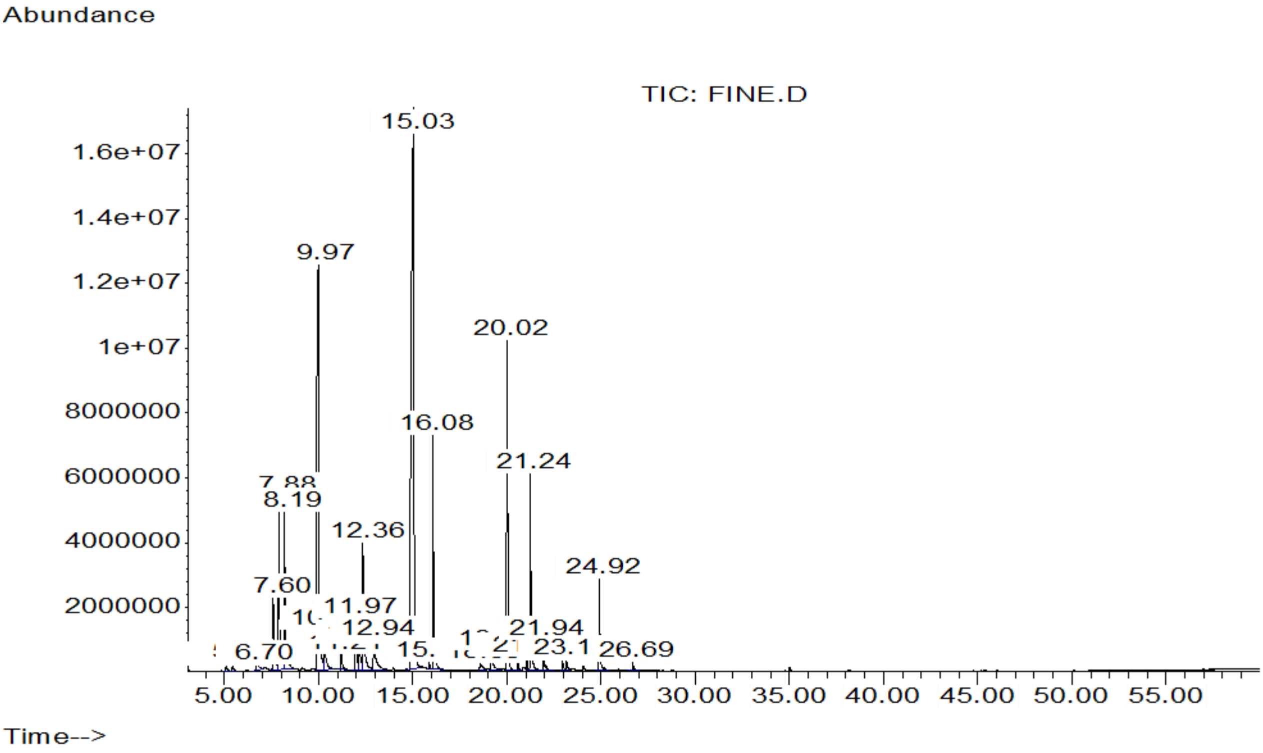

Figure S4. GC-MS chromatogram of essential oil from *L. angustifolia* variety 'Fine' (FINE)

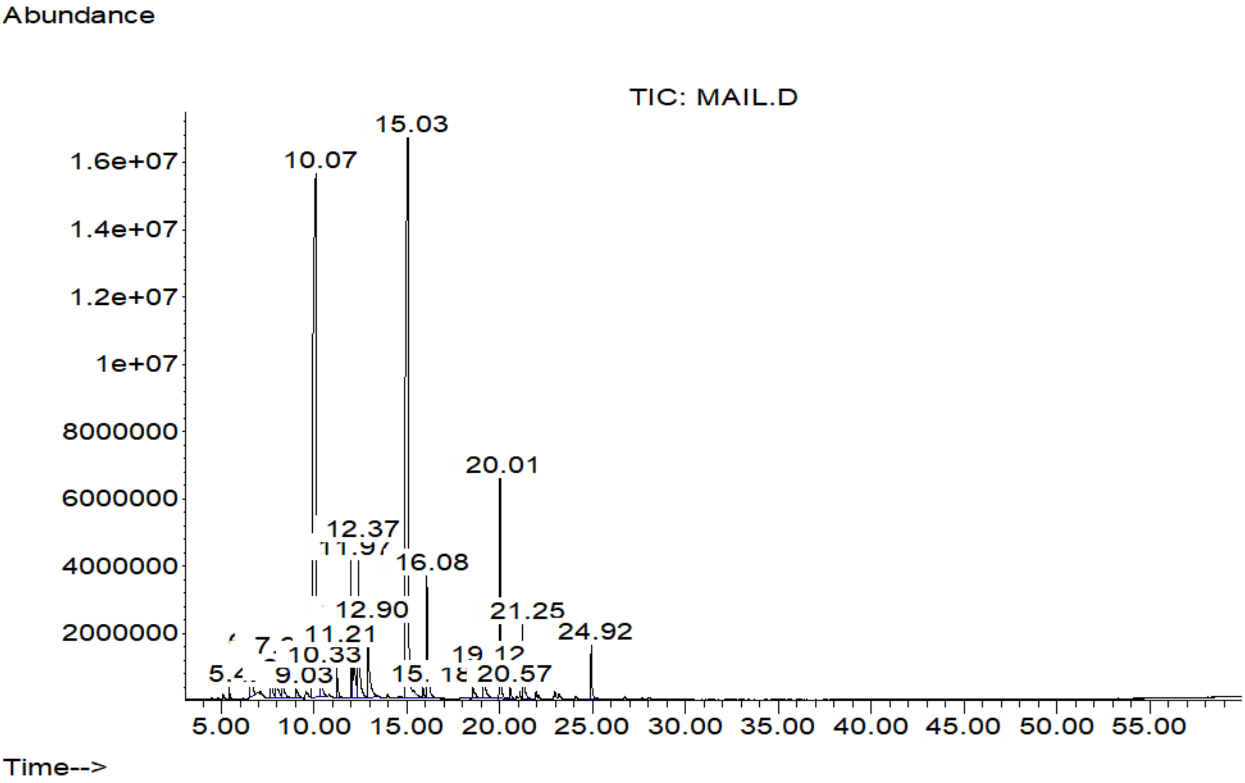

Figure S5. GC-MS chromatogram of essential oil from *L. angustifolia* variety 'Maillette' (MAIL)

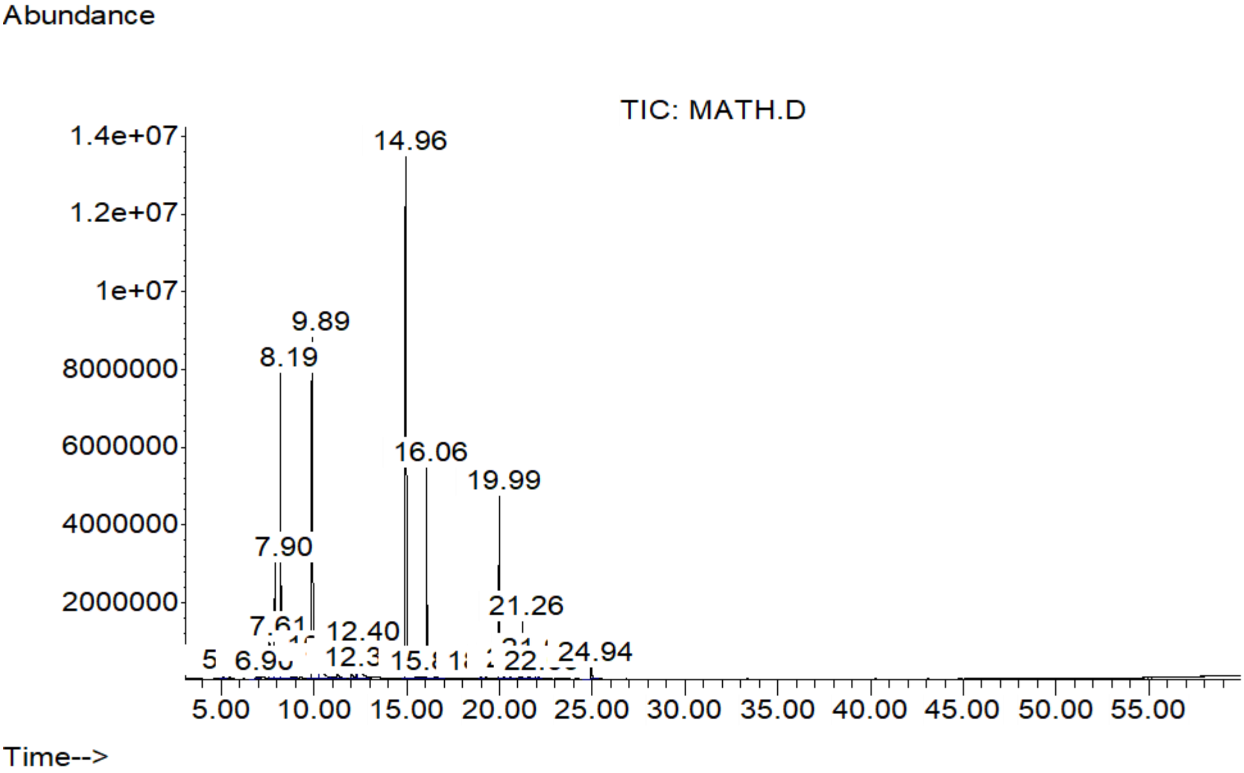

Figure S6. GC-MS chromatogram of essential oil from *L. angustifolia* variety 'Matherone' (MATH)

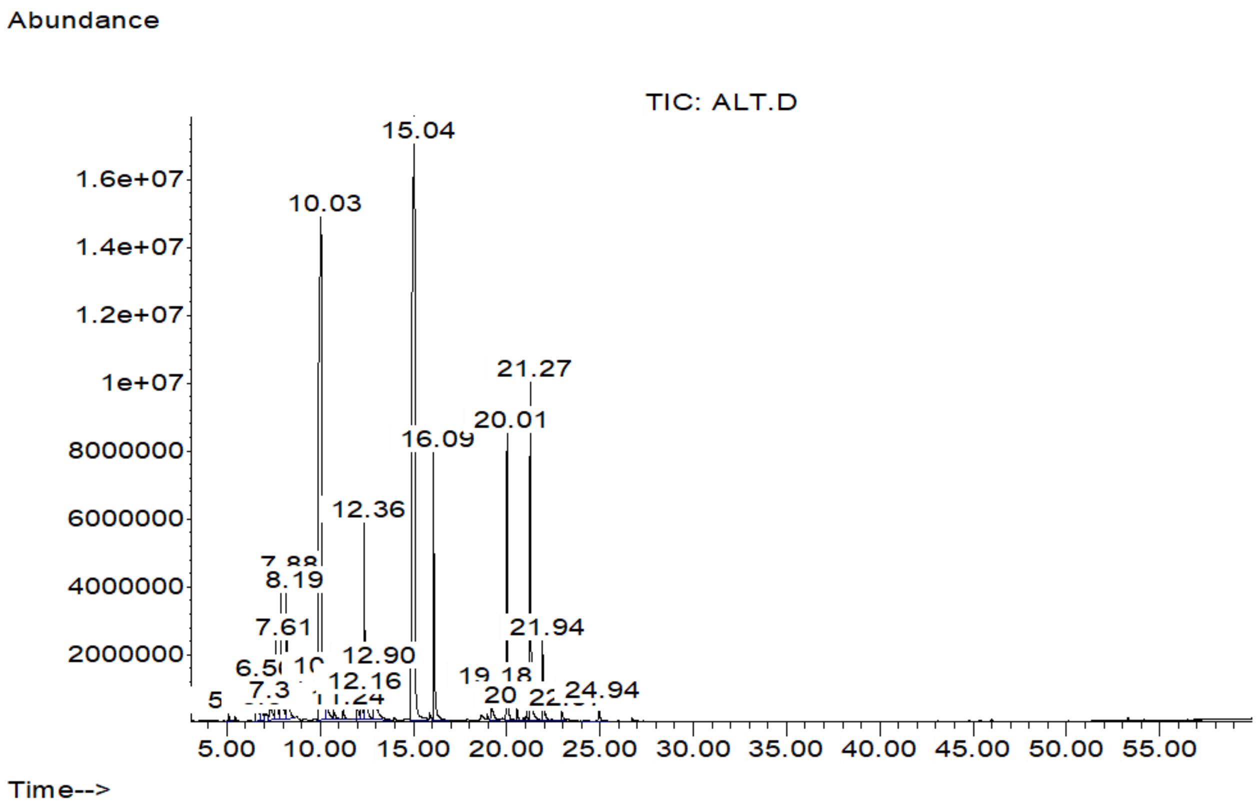

Figure S7. GC-MS chromatogram of essential oil from Bulgarian *L. angustifolia* produced by Alteya Organics (ALT)

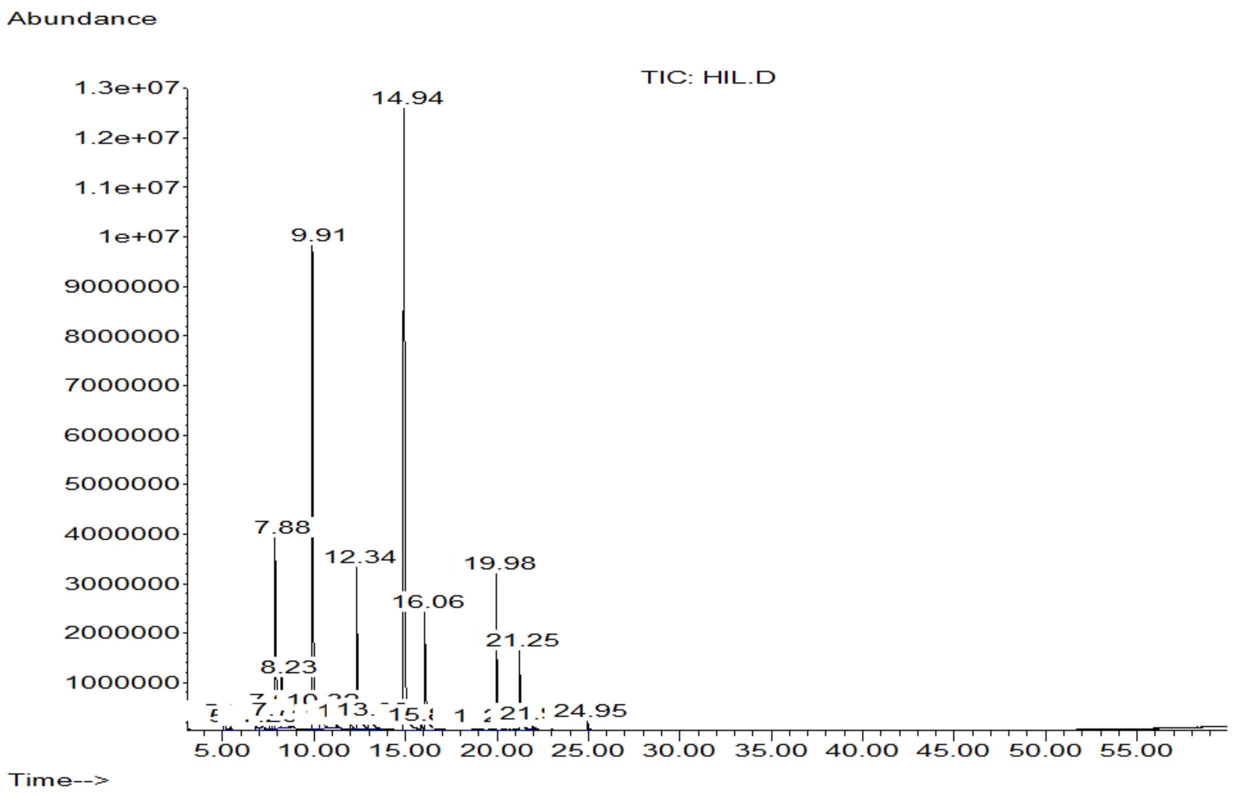

Figure S8. GC-MS chromatogram of essential oil from *L. angustifolia* variety 'Highland' (HIL)

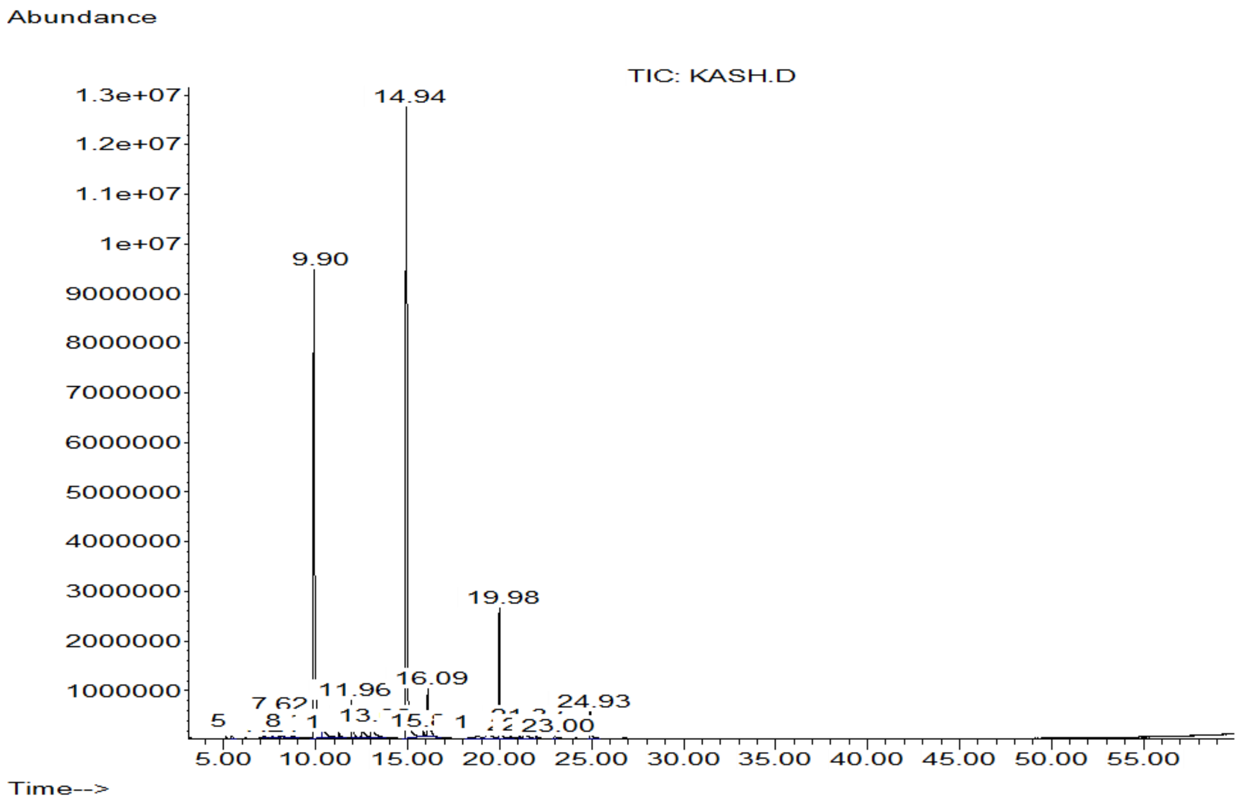

Figure S9. GC-MS chromatogram of essential oil from *L. angustifolia* variety 'Kashmir' (KASH)

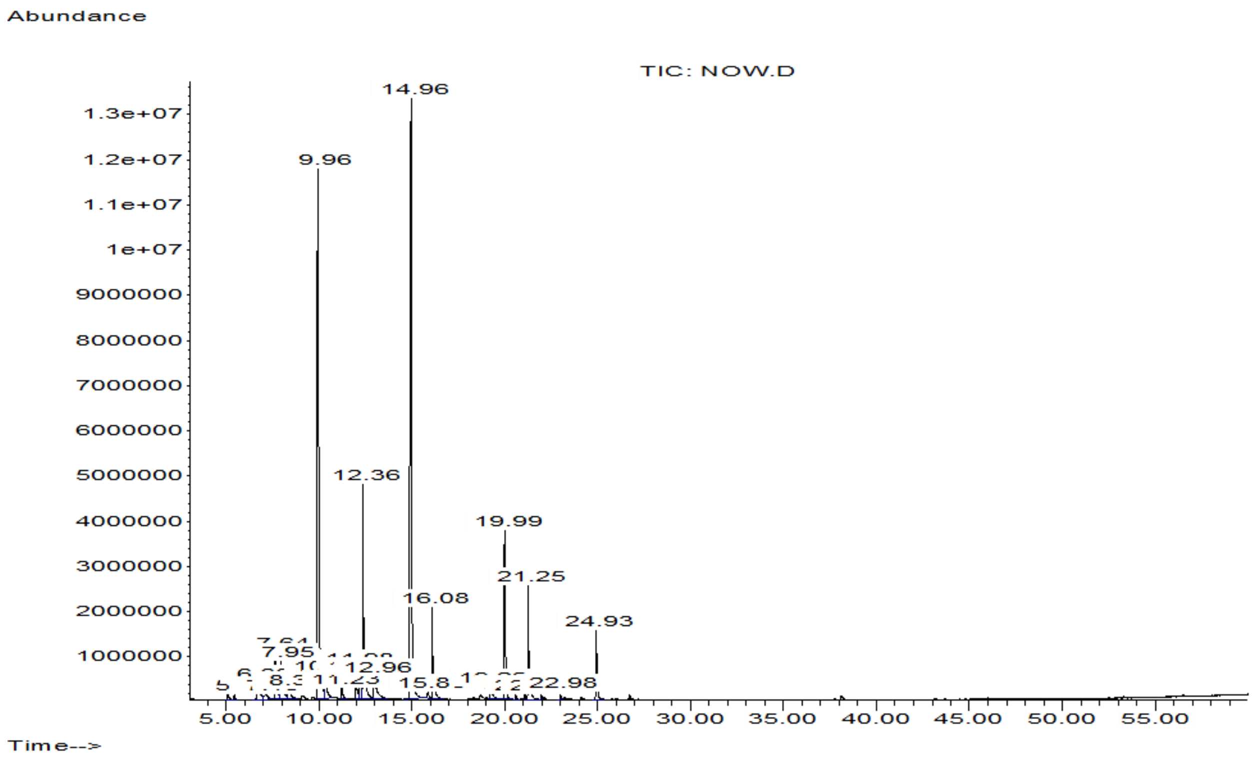

Figure S10. GC-MS chromatogram of essential oil from French *L. angustifolia* varieties produced by Now Foods (NOW)

(a)

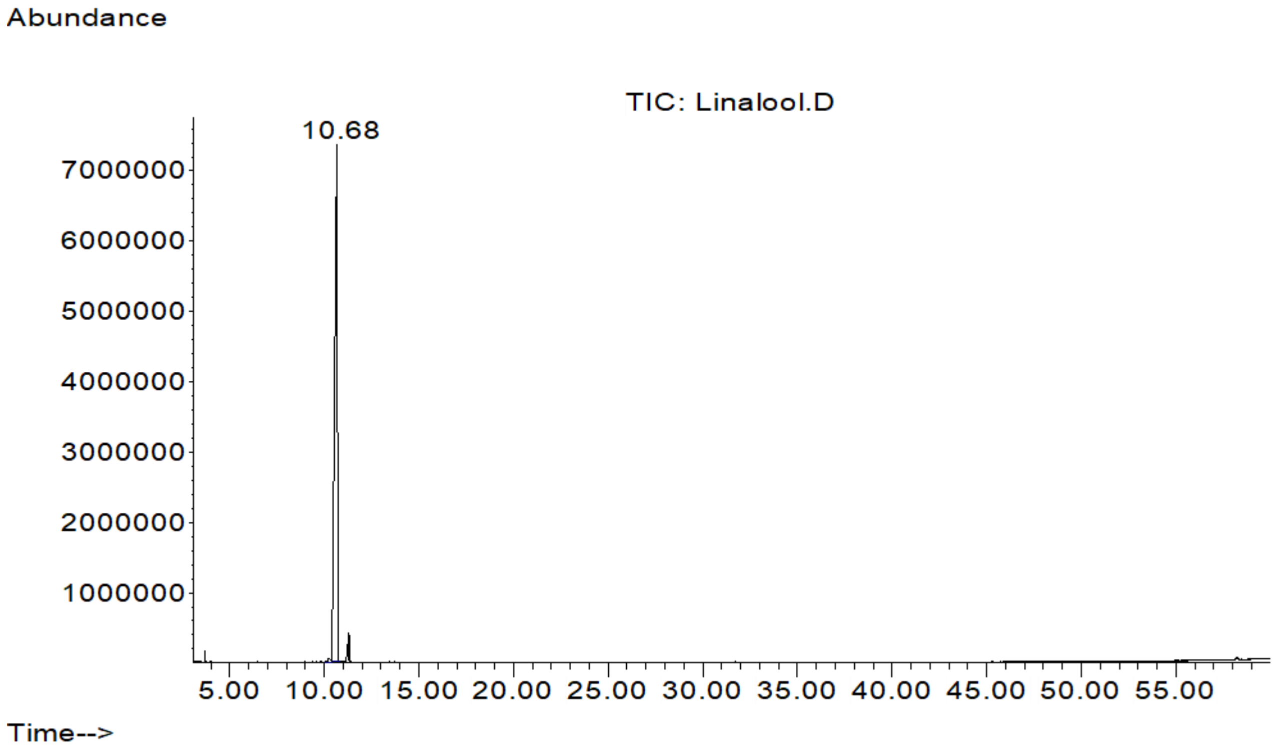

(b)

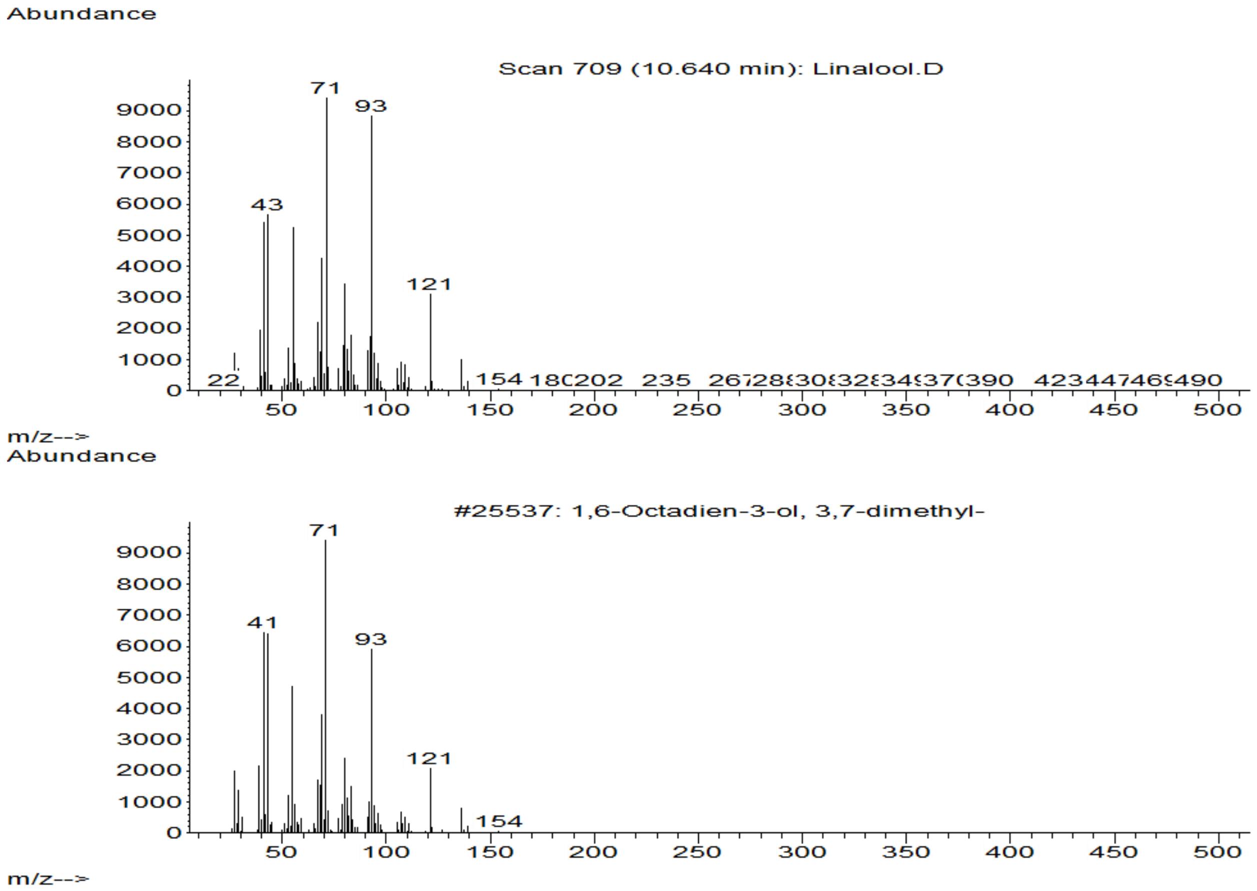

Figure S11. GC-MS chromatogram of linalool **(a)** and its mass spectrum compared with linalool standard mass spectrum from NIST 04 library **(b)**

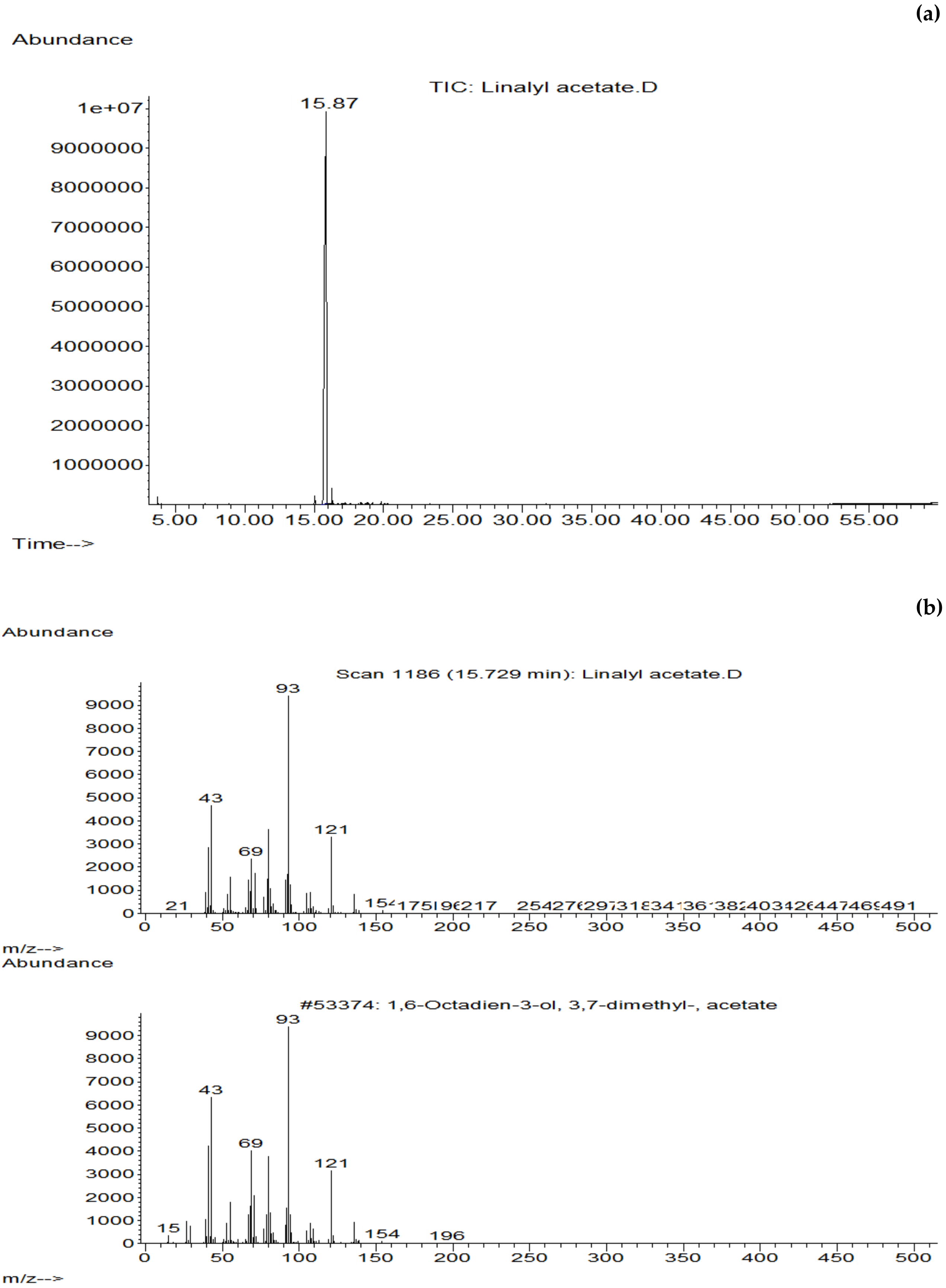

Figure S12. GC-MS chromatogram of linalyl acetate **(a)** and its mass spectrum compared with linalyl acetate standard mass spectrum from NIST 04 library **(b)**

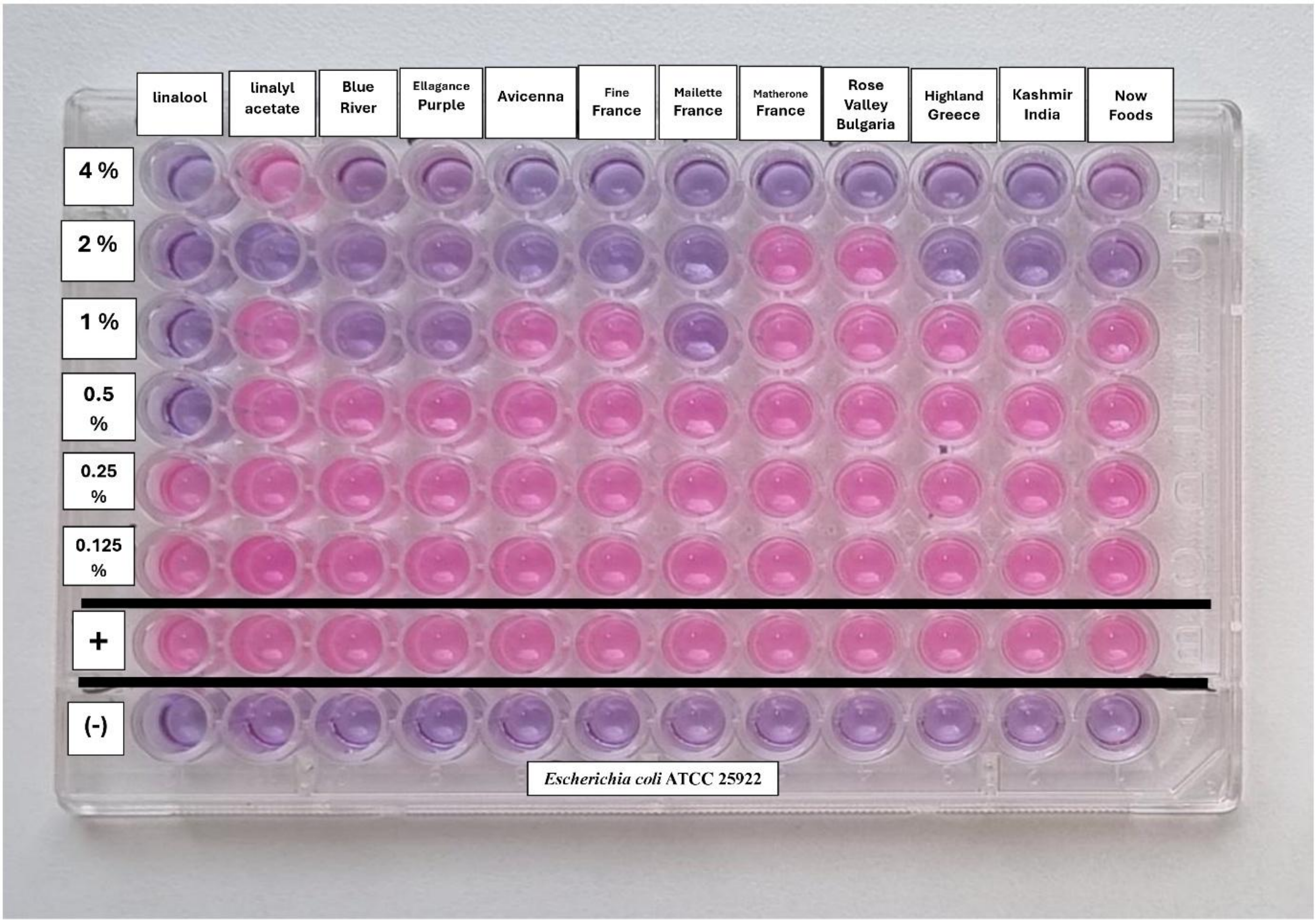

Figure S13. Example of plate for MIC determination after 24 hours of incubation and addition of resazurin for the antibiotic-sensitive *E. coli* ATCC 25922 strain
